# Cortical-blood vessel assembloids exhibit Alzheimer’s disease phenotypes by activating glia after SARS-CoV-2 infection

**DOI:** 10.1038/s41420-022-01288-8

**Published:** 2023-01-25

**Authors:** Dasom Kong, Ki Hoon Park, Da-Hyun Kim, Nam Gyo Kim, Seung-Eun Lee, Nari Shin, Myung Geun Kook, Young Bong Kim, Kyung-Sun Kang

**Affiliations:** 1 Adult Stem Cell Research Center and Research Institute for Veterinary Science, College of Veterinary Medicine, Seoul National University, Seoul, 08826 Republic of Korea; 2 Department of Research and Development, KR BIOTECH CO., Ltd., Seoul, 05029 Republic of Korea; 3 Department of Biomedical Science and Engineering, Konkuk Institute of Science and Technology, Konkuk University, Seoul, 05029 Republic of Korea

**Keywords:** Cell death and immune response, Tissue engineering, Acute inflammation

## Abstract

A correlation between COVID-19 and Alzheimer’s disease (AD) has been proposed recently. Although the number of case reports on neuroinflammation in COVID-19 patients has increased, studies of SARS-CoV-2 neurotrophic pathology using brain organoids have restricted recapitulation of those phenotypes due to insufficiency of immune cells and absence of vasculature. Cerebral pericytes and endothelial cells, the major components of blood-brain barrier, express viral entry receptors for SARS-CoV-2 and response to systemic inflammation including direct cell death. To overcome the limitations, we developed cortical-blood vessel assembloids by fusing cortical organoid with blood vessel organoid to provide vasculature to brain organoids a nd obtained the characteristics of increased expression of microglia and astrocytes in brain organoids. Furthermore, we observed AD pathologies, including β-amyloid plaques, which were affected by the inflammatory response from SARS-CoV-2 infection. These findings provide an advanced platform to investigate human neurotrophic diseases, including COVID-19, and suggest that neuroinflammation caused by viral infection facilitates AD pathology.

## Introduction

Since the global pandemic outbreak of COVID-19, severe acute respiratory syndrome coronavirus 2 (SARS-CoV-2) has infected over 608 million people with 6 million deaths by September 2022. Although the primary symptoms of COVID-19 are in respiratory system, increased case reports of neurological manifestations suggest that SARS-CoV-2 can directly infect or indirectly affect central nervous system [1]. In particular, brain infection with SARS-CoV-2 causes neuroinflammation, which can lead to various neurodegenerative diseases [2].

Neuroinflammation is an inflammatory response to protect central nervous system against infectious insults and injury [3]. Neuroinflammation is induced when the neurovascular unit, including microglia and astrocytes [4]. It has been reported that SARS-CoV-2 infects astrocytes/microglia and elicits proinflammatory activation which leads to neuronal death or dysfunction [5–7]. Furthermore, increased cytokines and glial cerebrospinal fluid markers in cerebrospinal fluid levels indicate glial activation after the inflammatory response in COVID-19 patients’ brain [8, 9].

Recently, neuroinflammation has been revealed as a key role in the pathogenesis of Alzheimer’s disease (AD) [10]. AD is a neurodegenerative disease with representative pathological hallmarks of β-amyloid (Aβ) plaques and hyperphosphorylated tau tangles [11, 12]. However, neurodegenerative mechanisms of AD, including amyloid cascade, are insufficient to explain the pathological processes [13]. The progressive activation of these glia with overproduction of proinflammatory cytokines subsequently proceeds Aβ deposition and causes neuronal cell death [14, 15]. In a recent study with the postmortem brain of COVID-19 patients, SARS-CoV-2 infection activates inflammatory signaling and oxidative stress pathways resulting in hyperphosphorylation of tau which is associated with AD. Furthermore, they reported some COVID-19 patients showed Alzheimer’s pathology [16].

Brain organoids are 3D self-assembled iPSC-derived model systems approximating fetal neocortex with gene expression profile resembling in vivo model compared to 2D cell cultures [17]. They have been used to study diseases with limitations in pathological observations because of their restricted accessibility [18]. Recently, developments in region-specific brain organoids have provided useful tools to investigate neurotropic diseases including COVID-19. [19, 20] However, unlike clinical reports mentioned above, the findings with region-specific brain organoids were partially restricted due to insufficiency of immune cells and the absence of vasculature might affect the results. hiPSC-derived brain organoids, including cortical organoids (COs), showed lower rates (<1.5%) of SARS-CoV-2 infection compared to choroid plexus organoids (10–20%), which expressed human SARS-CoV-2 receptor angiotensin-converting enzyme 2 (ACE2) [19]. Since ACE2 is mainly expressed in the blood-brain barrier [21], vascularized brain model is needed to overcome this low infection efficiency. In addition, in order to mimic the inflammatory response that occurs in the brain after infection, sufficient immune cells should exist within the organoids.

To overcome the limitations, we developed ‘cortical-blood vessel assembloids’ fused cortical-blood organoids (fCBOs) to provide 3D vasculature to brain organoids. After generation and characterization of COs and blood vessel organoids (BVOs), we cultured each organoid in the same well. Therefore, we obtained notable characteristics of increased expression of microglia/macrophages and astrocytes in brain organoids. Furthermore, we observed AD pathologies including Aβ plaques which were affected by the inflammatory response from SARS-CoV-2 infection.

## Results

### The fusion of cortical and blood vessel organoids

**Fig. 1:**
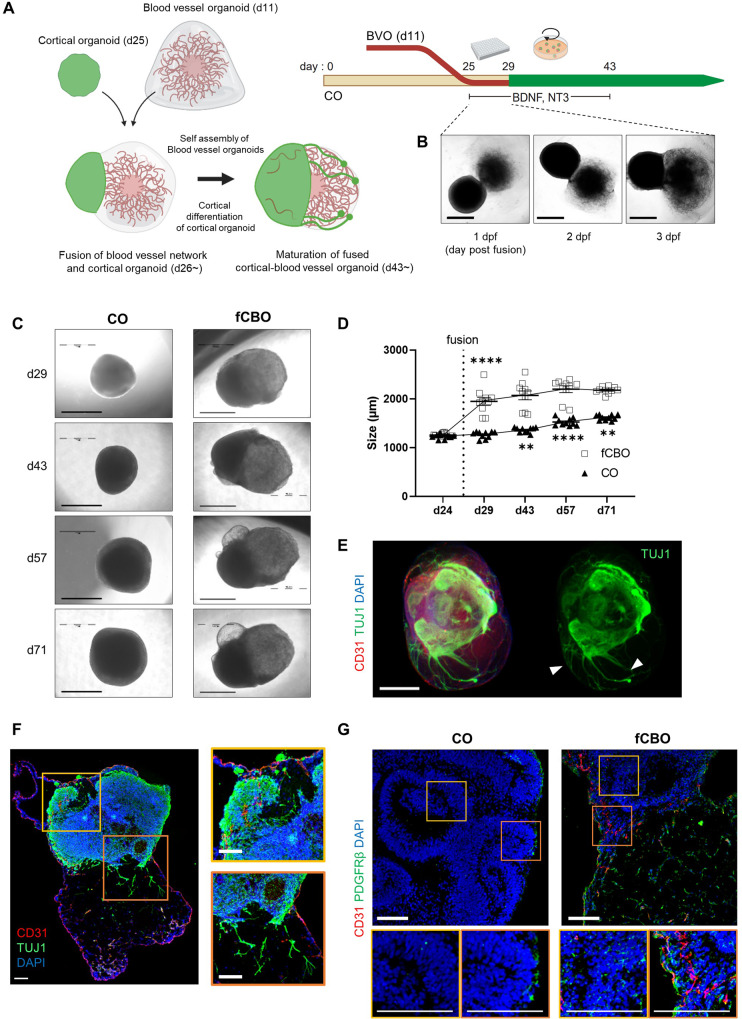
The fusion of cortical and blood vessel organoids. **A** Schematice of the method of the fusion of blood vessel organoids (BVOs) and cortical organoids (COs). **B** bright-field images of fused cortical-blood vessel organoids (fCBOs) during initial 3 days post fusion. Scale bars = 1 mm. **C** Bright-field images of COs and fCBOs on days 29, 43, 57 and 71 of cortical differentiation. Scale bars = 1 mm. **D** Quantification of the diameter during the 71 days of cortical differentiation (*n* = 10). **E** Whole-mount staining of fCBOs for endothelial marker CD31 and neuronal marker TUJ1 on day 43. Newly generated neurites (arrow). Scale bar = 100 μm. **F** Left, immunostaining of fCBOs for CD31 and TUJ1 (day 57). Penetrated blood vessels in cortical organoid region (yellow box), sprouted neurites to blood vessel organoid region (organge box). Scale bar = 100 μm. **G** Immunostaining of COs and fCBOs for pericyte marker PDGFRβ on day 57. Scale bar = 100 μm. Values represent mean with individual data points plotted. Error bars are SEM of the mean. *p < 0.05, **p < 0.01, ***p < 0.001 by unpaired two-sided *t*-test.

Before self-assembly of blood vessels, BVOs were embedded in matrix with collagen type 1 and Matrigel and showed pointed ends with sprouted blood vessels. During cortical differentiation of CO, BVOs undergo self-assembly (Fig. S1A). Vascular structure of BVOs consists of endothelial cells (ECs), vascular smooth muscle cells, pericytes and basement membrane (Fig. S1B). To generate cortical-blood vessel assembloids, we fused COs (d25) with BVOs (d11) during cortical differentiation (Fig. 1A). While the deep layers are being developed, BVO and CO are put together in a U-bottom well and allowed to formulate physical fusion for 3 days. During first 3 days of fusion, organoids in the same well became fused with each other (Fig. 1B) with the yield of 98.3% (118/120 succeed) and fused organoids were transferred and cultured under spinning condition. The fusion of two or three BVOs with a single CO were also possible when the organoids were placed in a single well (Fig. S1C). However, we decided to use fCBOs; with the ratio of 1:1 CO and BVO, to reduce morphological variations.

The morphological changes in fCBOs were observed in both cortical and blood vessel regions. (Fig. 1C). After fusion at day 25, the growth rates of fCBOs and COs were increased similarly until day 57 (Fig. 1D). On day 43, fCBOs expressed endothelial marker CD31 and neuronal marker TUJ1 and showed newly generated neurites directed to blood vessel region (Fig. 1E). The neurites expressed TUJ1 but were negative for mature neuronal marker MAP2 (Fig. S1D). In fCBOs, endothelial tubes penetrated to cortical region and cysts generated nearby cortical regions were observed (Fig. 1F). Moreover, fCBOs expressed PDGFRβ^+^ pericytes in the cortical region, whereas COs rarely contain in their inner deep layers (Fig. 1G). We examined the perfusability of blood vessels in fCBOs by assessing capillary networks. FITC-dextran was detected in CD31 positive vasculature in blood vessel regions of fCBO, whereas COs did not (Fig. S1F, G). In these results, fCBOs attain vascularized cortical regions which also includes pericytes and as an improved CO model by obtaining functional BVOs.

### Fused cortical-blood vessel organoids with glial expression

To characterize the neuronal subtypes in cortical region, we confirmed layer-specific cortical neurons by immunostaining for MAP2, TBR1, CTIP2, SATB2, PAX6 and TBR2 in fCBOs and COs (Fig. S2A). Blood-brain barrier is formed by microvascular ECs lining the cerebral capillaries penetrating the brain [22]. The expression of tight junctions including ZO1, claudin-5 between adjacent ECs preventing the unregulated molecular passage [23]. We noted that fCBOs exhibited blood brain barrier-like characteristics with tight junctions and adherens junctions (Fig. S2B). ZO1 expressed within lumens and around blood vessels penetrated cortical region. In addition, claudin-5 and β-catenin were costained with vascular networks.

**Fig. 2:**
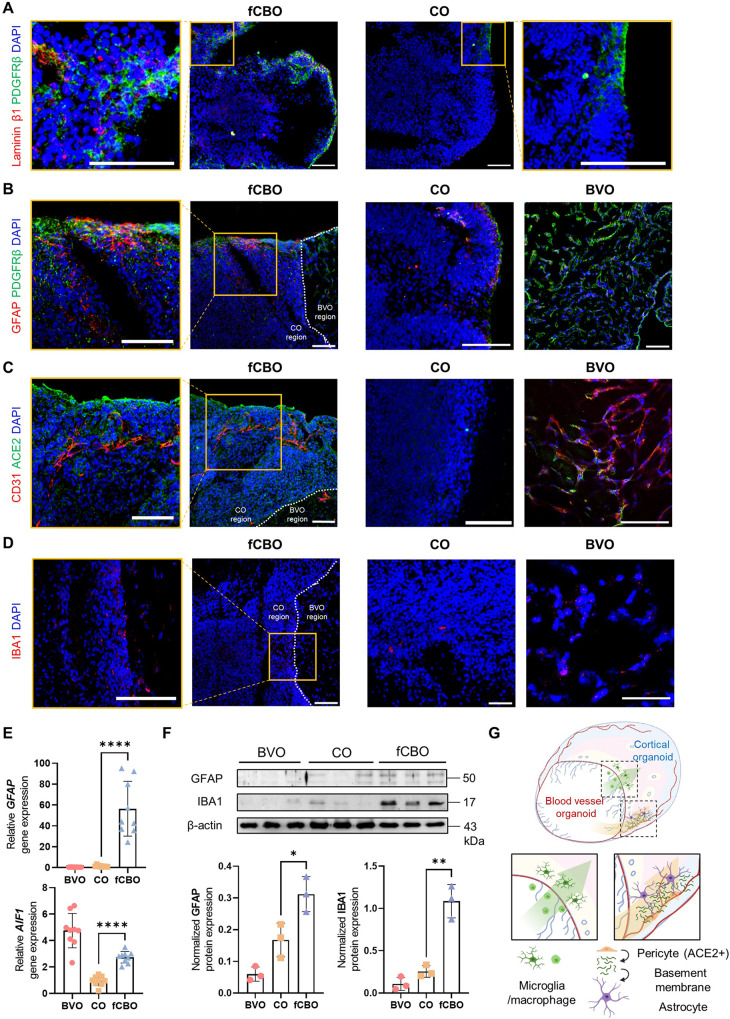
Fused cortical-blood vessel organoids with glial expression. **A** Immunostaining of fCBOs and COs for laminin-β1 and pericyte marker PDGFRβ on day 57. Scale bar = 100 μm. **B** Immunostaining of fCBOs (day 57), COs (day 57), and BVOs (day 15) for astrocyte marker GFAP and pericyte marker PDGFRβ. Scale bar = 100 μm. **C** Immunostaining of fCBOs (day 57), COs (day 57), and BVOs (day 15) for SARS-CoV-2 receptor ACE2 and CD31 on day 57. Scale bar = 100 μm. **D** Immunostaining of fCBOs (day 57), COs (day 57), and BVOs (day 15) for microglia/macrophage marker IBA1 on day 57. Scale bar = 100 μm. **E** Relative GFAP and AIF1 gene levels in BVOs (day 15), COs (day 57) and fCBOs (day 57) (*n* = 9). **F** Relative GFAP and IBA1 protein levels in BVOs (day 15), COs (day 57) and fCBOs (day 57) (*n* = 3). **G** Schematic image of fCBO. Values represent mean with individual data points plotted. Error bars are SEM of the mean. *p < 0.05, **p < 0.01, ***p < 0.001 by unpaired two-sided *t*-test.

We noted that laminin-β1 was expressed around pericytes in fCBOs, which is absent from COs (Fig. 2A). fCBOs expressed astrocyte marker GFAP adjacent to pericytes, whereas COs rarely expressed GFAP and BVOs did not contain astrocytes (Fig. 2B). Furthermore, ACE2 was expressed in fCBOs and BVOs, but little in COs (Fig. 2C). These ACE2 expression might be attributed to pericytesoriginated from BVOs, which synthesized basement membrane promoting differentiation and maturation of astrocytes [24, 25]. Microglia/macrophage marker IBA1 was expressed in blood vessel region and around the fused border. In BVOs, we observed IBA1^+^ cells around endothelial tubes, which surrounded S100^+^ MSC-like cells, and COs rarely expressed IBA1 (Fig. 2D). To further characterize IBA1^+^ cells in fCBOs, we performed flow cytometry after labeling with CD34, CD14 and CD11b. fCBO presented ~5% CD34^+^CD14^-^ hematopoietic cells and 1.4% CD14^+^CD34^-^ cells. Most of CD14^+^ cells in fCBO were positive in CD11b, which is a marker of microglia/macrophages. These cell populations were also presented in BVO (Fig. S2C). Relative expression of glial marker genes and proteins in BVOs, COs and fCBOs revealed that fCBO expressed significantly increased glial cells than COs (Fig. 2E, F).

Together, fCBOs presented microglia/macrophages and pericytes from BVOs and upregulated differentiation of astrocytes compared to COs (Fig. 2G). These data indicate that fCBOs which express ACE2 could be a suitable model for SARS-CoV-2 neurotrophic studies containing microglia/macrophages and astrocytes.

### SARS-CoV-2 infects fused cortical-blood vessel organoids and induced neuronal cell death and microvascular injury

**Fig. 3:**
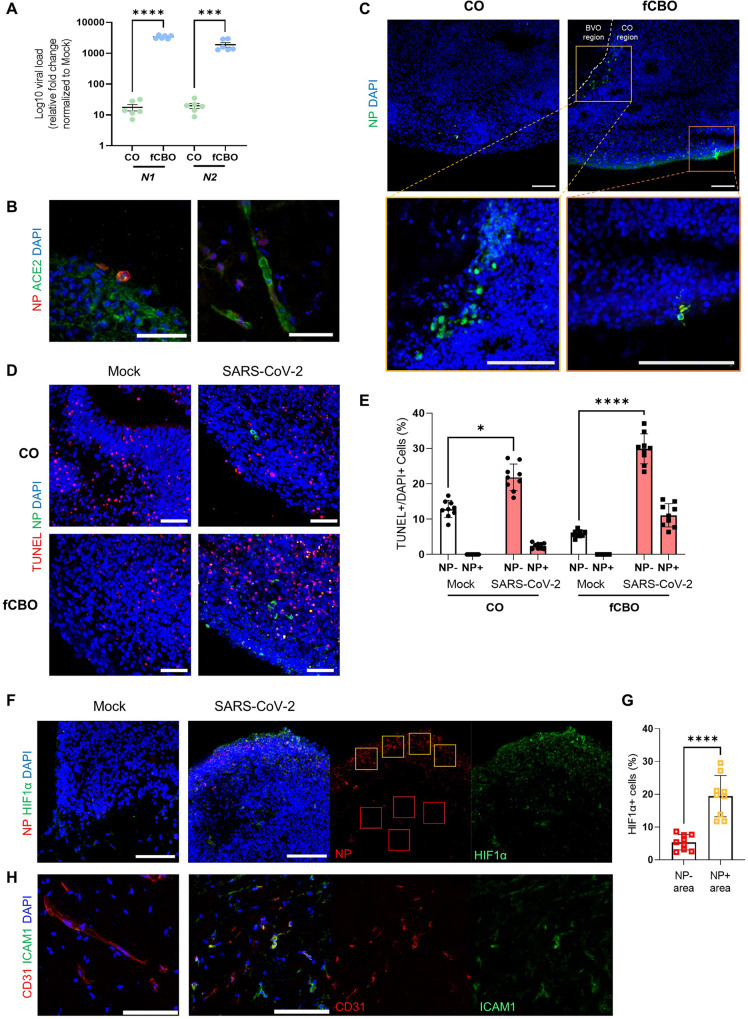
SARS-CoV-2 infects fused cortical-blood vessel organoids. **A** Relative viral RNA levels for SARS-CoV-2 N1 and N2 gene of SARS-CoV-2 infected COs and fCBOs normalized to mock controls by qRT-PCR (*n* = 6). **B** Immunostaining of SARS-CoV-2 infected fCBOs for SARS-CoV-2 nucleocapside protein (NP) and ACE2 on day 57. Cortical organoid region (left) and blood vessel organoid region (right). Scale bars = 50 μm. **C** Immunostaining of SARS-CoV-2 infected COs and fCBOs for NP in ventricular zone (yellow box) and superficial layer (orange box). Scale bar = 100 μm. **D** Immunostaining of SARS-CoV-2 or mock infected COs and fCBOs for TUNEL and NP. Scale bars = 100 μm. **E** The percentage of NP^-^ or NP^+^TUNEL^+^ cells in DAPI^+^ cells of SARS-CoV-2 or mock infected COs and fCBOs (*n* = 5 organoids with 4 images per organoid). **F** Immunostaining of SARS-CoV-2 or mock infected fCBOs for NP and hypoxia marker HIF1α. Scale bars = 100 μm. **G** The percentage of HIF1α^+^ cells in NP^-^ (red boxes) or NP^+^ (yellow boxes) area of SARS-CoV-2 infected fCBOs (*n* = 5 fCBOs, 4 areas per organoid). **H** Immunostaining of SARS-CoV-2 or mock infected fCBOs for CD31 and activated endothelial marker ICAM1. Scale bars = 100 μm. Values represent mean with individual data points plotted. Error bars are SEM of the mean. *p < 0.05, **p < 0.01, ***p < 0.001 by unpaired two-sided *t*-test.

To examine the susceptibility of fCBO to SARS-CoV-2 infection and investigate neurotropism, we incubated fCBOs with SARS-CoV-2 and then harvested at 4-days post-infection (dpi) for analysis. To induce severe infection, viral titers at 2 × 10^6^ TCID_50_/ml were used based on N1 gene expression level (Fig. S3A). SARS-CoV-2 nucleocapside protein (NP) and N1, N2 gene were significantly overexpressed in infected fCBOs than infected COs (Figs. 3A and S3B). Costained NP and ACE2 in cortical region and blood vessel region indicated viral entry via ACE2 (Fig. 3B). Infected cells in the cortical region were mainly presented at phospho-vimentin^+^ VZ or TUJ1^+^ superficial layers (Figs. 3C and S3C). In addition, infected cells, which costained with CD31, were observed in blood vessel region (Fig. S3D).

SARS-CoV-2 induces neuronal cell death and a locally hypoxic environment in brain organoids [26]. These findings were also observed in fCBOs via a TUNEL assay. Infected fCBOs showed increase of TUNEL^+^ cells compared to mock control, however, increase in the number of TUNEL^+^ cells in COs was less than fCBOs after infection (Fig. 3D). The number of TUNEL^+^ cells was significantly increased in infected fCBOs and most TUNEL^+^ cells were NP negative (Fig. 3E). This uninfected cell death occurred adjacent to infected cells. Indirect effects of SARS-CoV-2 infection can occur via induced hypoxia [27]. We stained HIF1α and observed that fCBOs expressed HIF1α nearby infected cells (Fig. 3F). Quantification of HIF1α^+^ cells in NP^+^ area was significantly increased than NP^-^ area (Fig. 3G). In addition, Hif1α pathway genes including HIF1A, ARNT and SCL2A1 were significantly upregulated in infected fCBOs (Fig. S3E). Due to the hypoxic effect and inflammatory responses on ECs [28, 29], activation of ECs led to ICAM1 and VCAM1 expression in infected fCBOs (Fig. 3H). Moreover, we observed that infected ECs exhibited fragmented and condensed cell shapes implying cell damage compared to structure of the endothelial tubes of the mock control (Fig. S3F). Infected fCBOs reproduced progeny virus after infection (Fig. S3G). Thus, local hypoxic environment induced by SARS-CoV-2 infection activated ECs and induced adjacent cell death in infected fCBOs.

### SARS-CoV-2 infected fused cortical-blood vessel organoids exhibit Alzheimer’s disease pathology

**Fig. 4:**
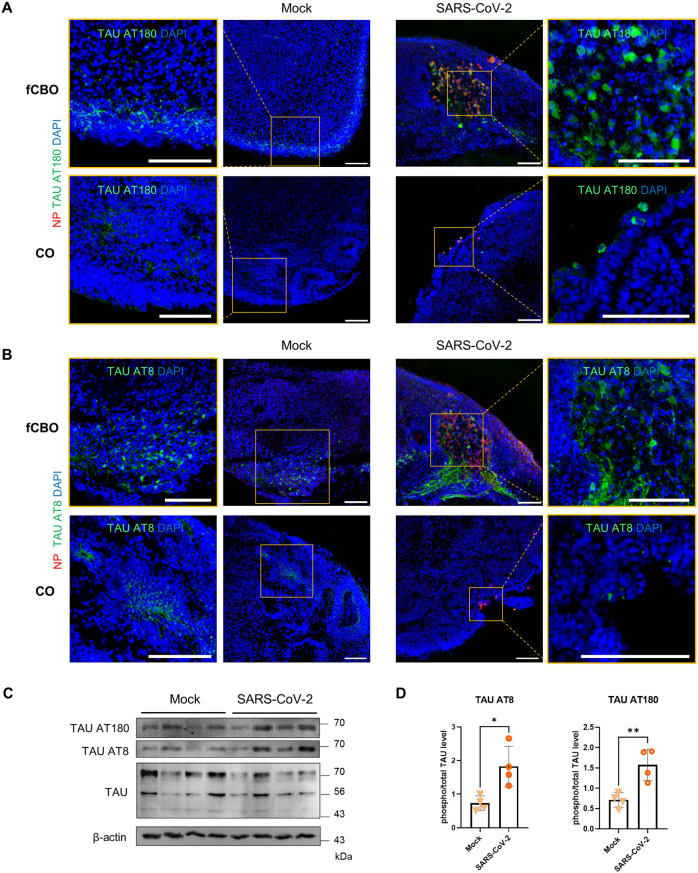
SARS-CoV-2 infected fused cortical-blood vessel organoids expressed hyperphosphorylated tau. **A, B** Immunostaining of SARS-CoV-2 or mock infected fCBOs and COs for NP and tau phosphorylation marker AT180 (pT231Tau) (**A**) and AT8 (pS202T205Tau) (**B**). Scale bars = 100 μm. **C** Western blot of SARS-CoV-2 or mock infected fCBOs for AT180, AT8, TAU and the loading control β-actin. **D** Phospho/total TAU protein levels in SARS-CoV-2 or mock infected fCBOs for AT180 and AT8 (*n* = 4). Values represent mean with individual data points plotted. Error bars are SEM of the mean. *p < 0.05, **p < 0.01, ***p < 0.001 by unpaired two-sided *t*-test.

Aberrant Tau localization with phospho-Tau (pTau) expression in SARS-CoV-2 infected neurons of brain organoids was observed in recent study [30]. This mislocalization was also observed in fCBO and the expression of phenotype was increased due to the higher infection rate than COs. Compared to mock control, infected fCBOs exhibited tau phosphorylation marker AT180 (pT231Tau) and AT8 (pS202T205Tau) in the infected cells. However, the expression of AT180 and AT8 was low in infected COs (Fig. 4A, B). Although this expression of pTau was mostly in soma of infected cells. Although AT8 (pS202T205Tau) was mostly expressed in axons, some infected cells exhibited AT8 at soma (Fig. 4B). Protein expression levels of pTau was also upregulated in infected fCBOs (Fig. 4C). Moreover, pTau/total Tau expression levels were significantly increased after infection (Fig. 4D).

**Fig. 5:**
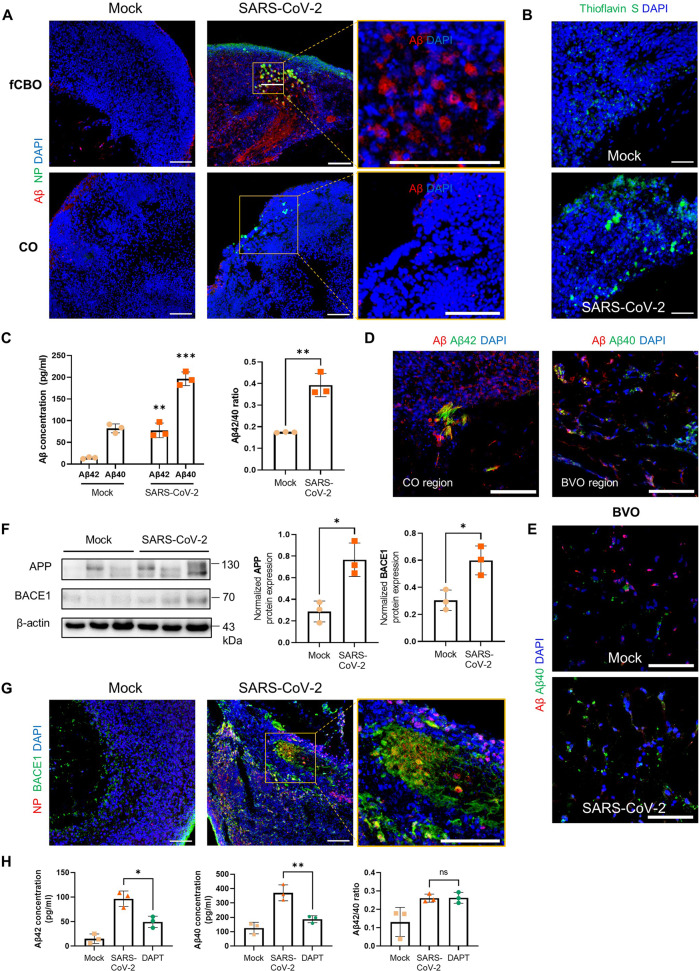
SARS-CoV-2 infected fused cortical-blood vessel organoids expressed β-amyloid plaques. **A** Immunostaining of SARS-CoV-2 or mock infected fCBOs and COs for NP and Aβ. Scale bars = 100 μm. **B** Thioflavin-S staining of SARS-CoV-2 or mock infected fCBOs. Scale bars = 100 μm. **C** Concentrations of Aβ42 and Aβ40 quantified by ELISA and Aβ peptide 42/40 ratios in fCBOs before and after SARS-CoV-2 infection (*n* = 3). **D** Immunostaining of SARS-CoV-2 infected fCBOs for Aβ and 42-residue Aβ (Aβ42) or 40-residue Aβ (Aβ40). Cortical organoid region (left) and blood vessel organoid region (right). Scale bars = 100 μm. **E** Immunostaining of SARS-CoV-2 or mock infected BVOs for Aβ and Aβ40. Scale bars = 50 μm. **F** Western blot for β-amyloid precursor protein (APP), β-secretase (BACE1) and the loading control β-actin of SARS-CoV-2 or mock infected fCBOs and relative protein levels. (*n* = 3). **G** Immunostaining of SARS-CoV-2 or mock infected fCBOs for BACE1 and NP. Scale bars = 100 μm. **H** Concentrations of Aβ42 and Aβ40 quantified by ELISA and Aβ peptide 42/40 ratios in mock control, SARS-CoV-2 infected and DAPT treated fCBOs (*n* = 3). Values represent mean with individual data points plotted. Error bars are SEM of the mean. *p < 0.05, **p < 0.01, ***p < 0.001 by unpaired two-sided *t*-test.

Since aberrantly phosphorylated Tau expression is shown during the early stages of Tau pathology, which is the key phenotype of AD, we consequently investigated another phenotype of AD; Aβ pathology. We performed immunostaining for Aβ in fCBOs and COs. Aβ accumulations, another main hallmark of AD, was found in SARS-CoV-2 infected fCBOs (Fig. 5A). Aβ plaques were confirmed with Thioflavin-S staining (Fig. 5B). Processed Aβ species during in the amyloidogenic pathway, 42-residue Aβ (Aβ42) shows a significant increase in AD [31]. Because of their hydrophobic amino acids compared to 40-residue Aβ (Aβ40), Aβ42 promotes fibril formation which could be induced neurotoxic effects [32]. In contrast, Aβ40 is the predominant Aβ species in vascular deposit [33]. In ELISA analysis of fCBO lysis, we observed that concentrations of Aβ42 and Aβ40 were significantly increased after infection and Aβ42/Aβ40 ratio was also upregulated in infected fCBOs (Fig. 5C). Furthermore, Aβ42 was expressed in fibril forms, and Aβ40 was expressed in blood vessel region (Fig. 5D). In COs and BVOs, Aβ expressions were upregulated after infection but no plaque was detected (Fig. 5E). Aβ is generated from β-amyloid precursor protein (APP) through cleavages by two enzymes including β-secretase (BACE1) and γ-secretase complex [34]. After infection, APP and BACE1 expression levels of fCBO were increased whereas γ-secretase complex proteins including PSEN1/2 levels were not (Figs. 5F and S4A). We stained BACE1 in infected and mock fCBOs and found that BACE1 overexpression was adjacent with SARS-CoV-2 infected cells (Fig. 5G). BACE1 expression after infection was also increased in COs, but the expression levels were lower than fCBOs (Fig. S4B). Moreover, gamma secretase inhibitor DAPT treatment, at the concentration of 10 μM, decreased concentration of both Aβ42 and Aβ40 after infection, but Aβ42/40 ratio did not change (Fig. 5H). Altogether, SARS-CoV-2 infeced fCBOs exhibited AD pathologies, including hyperphosphorylated tau and amyloid plaques.

### Fused cortical-blood vessel organoids show upregulated glial expression after SARS-CoV-2 infection

**Fig. 6:**
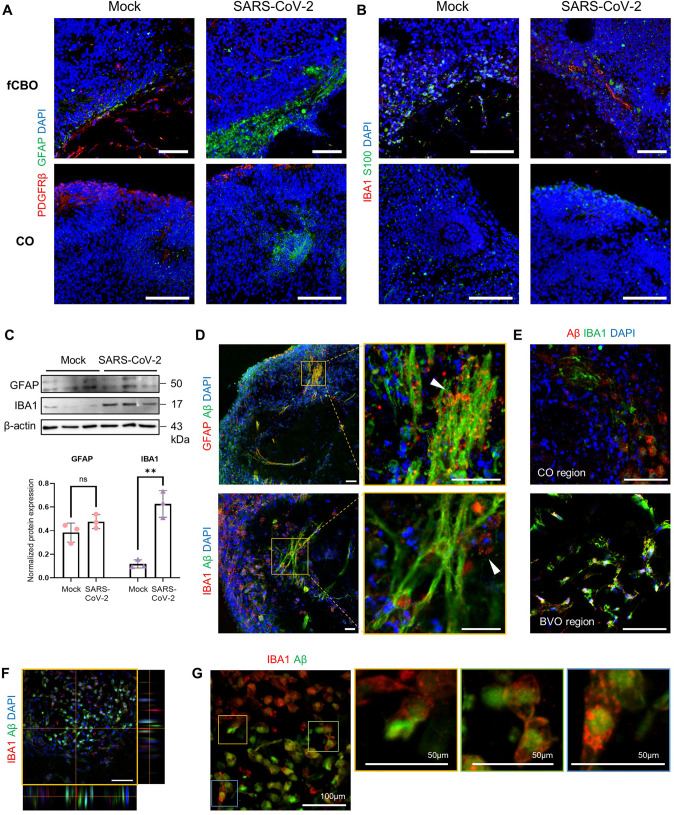
Recruitment of microglia/macrophages and astrocytes after SARS-CoV-2 infection in fused cortical-blood vessel organoids. **A** Immunostaining of SARS-CoV-2 or mock infected fCBOs and COs for GFAP and PDGFRβ. Scale bar = 100 μm. **B** Immunostaining of SARS-CoV-2 or mock infected fCBOs and COs for IBA1 and S100. Scale bar = 100 μm. **C** Western blot for GFAP, IBA1 and the loading control β-actin of SARS-CoV-2 or mock infected fCBOs and relative protein levels (*n* = 3). **D** Immunostaining of SARS-CoV-2 infected fCBOs for Aβ and GFAP or IBA1. Accumulated astrocytes (upper arrow) or microglia/macrophages (lower arrow) around Aβ plaques. Scale bar = 100 μm. **E** Immunostaining of SARS-CoV-2 infected fCBOs for Aβ and IBA1. Ramified microglia accumulated in Aβ^+^ cells in cortical organoid region and blood vessel organoid region. Scale bars = 50 μm. **F** Representative Z-stack image of immunostaining of SARS-CoV-2 infected fCBOs for IBA1 and Aβ. IBA1 + microglia/macrophages accumulated around Aβ + cells. **G** Zoom-in image of glial phagocytosis of Aβ + cells (yellow, green and blue boxes). Values represent mean with individual data points plotted. Error bars are SEM of the mean. *p < 0.05, **p < 0.01, ***p < 0.001 by unpaired two-sided *t*-test.

Immune cells including glial cells (especially microglia and astrocytes) and macrophages drive neuroinflammation contributing to the progression of AD [35, 36]. Notably, we observed that glial expression in organoids was significantly increased after SARS-CoV-2 infection (Fig. 6A). Especially, IBA1 expression was markedly upregulated in infected fCBO (Fig. 6B, C). Furthermore, these glial cells were recruited to Aβ plaques. Astrocytes were found closely associated with Aβ plaques and IBA1^+^ microglia/macrophages also surrounded Aβ plaques (Fig. 6D). Ramified IBA1^+^ cell accumulation was detected in Aβ^+^ cells in cortical organoid region and blood vessel organoid region (Fig. 6E). Z-stack of confocal image showed that these phagocytic IBA1^+^ cells were presented nearby Aβ^+^ cells (Fig. 6F) and we could find macrophage/microglial phagocytosis in infected fCBOs (Fig. 6G). However, compared to fCBOs, changes of glial expression in COs and BVOs were insignificant (Fig. S4C). In these results, the glial population of fCBO increased due to SARS-CoV-2 infection, and in particular, it was recruited around the accumulated Aβ plaque which is the pathology of AD.

### Proinflammatory cytokines induce the activation of microglia and astrocytes in SARS-CoV-2 infected fused cortical-blood vessel organoids

**Fig. 7:**
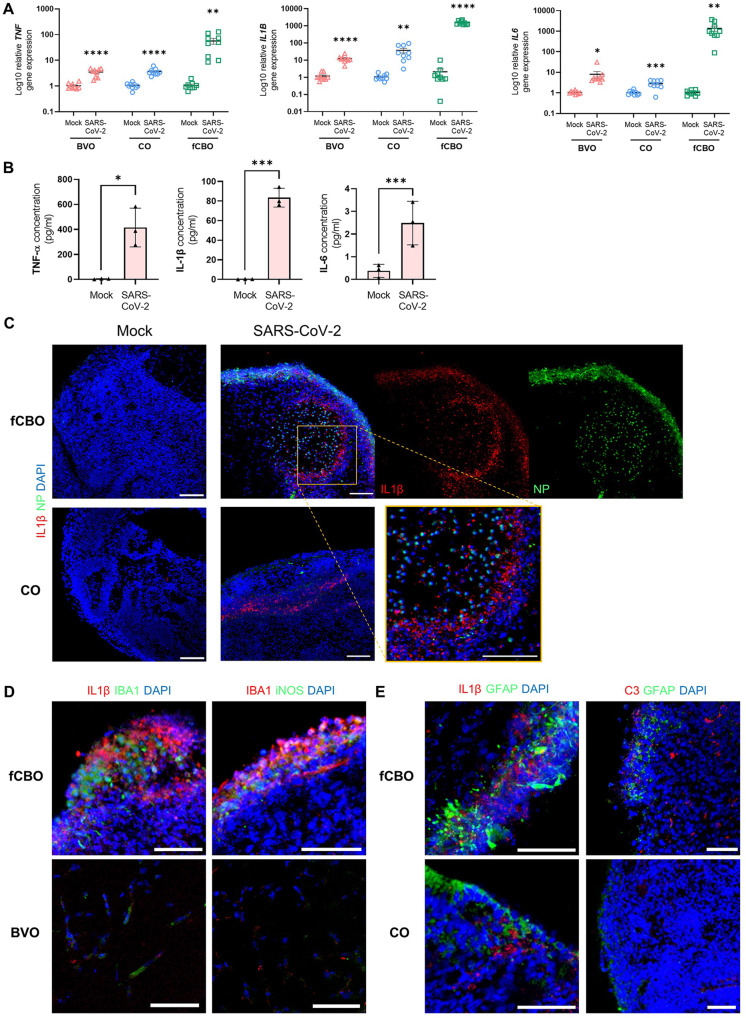
Proinflammatory cytokines induce the activation of microglia and astrocytes in SARS-CoV-2 infected fused cortical-blood vessel organoids. **A** Relative gene expression of SARS-CoV-2 or mock infected BVOs, COs and fCBOs for TNF, IL1B and IL6 (*n* = 9). **B** Quantification of released proinflammatory cytokines, TNFα, IL1β and IL6, from SARS-CoV-2 or mock infected fCBOs by ELISA (*n* = 3). **C** Immunostaining of SARS-CoV-2 or mock infected fCBOs and COs for IL1β and NP. Scale bars = 100 μm. **D** Immunostaining of SARS-CoV-2 infected fCBOs and BVOs for IL1 β and IBA1 (left) and activated glial marker iNOS and IBA1 (right). Scale bars = 100 μm. **E** Immunostaining of SARS-CoV-2 infected fCBOs for IL1β and GFAP (left) and activated astrocyte marker C3 and GFAP (right). Scale bars = 100 μm. Values represent mean with individual data points plotted. Error bars are SEM of the mean. *p < 0.05, **p < 0.01, ***p < 0.001 by unpaired two-sided *t*-test.

As it has been recently reported that SARS-CoV-2 triggers inflammatory responses via a pyroptosis of host cells that release damage-associated molecules [37, 38], we performed RT-PCR analysis for proinflammatory cytokine genes and ELISA analysis of secreted proinflammatory cytokines including TNFα, IL1β and IL6, in infected fCBOs. TNFα, IL1β and IL6 are known to be associated with post-acute sequelae of COVID-19 [39] and used as biomarkers of SARS-CoV-2 infection in previous study [40]. In RT-PCR analysis, an increase of TNF, IL1B and IL6 genes was also found in COs and BVOs, but the level of increase was less than fCBOs (Fig. 7A). All expression levels of secreted proinflammatory cytokines were significantly increased relative to mock control fCBOs (Fig. 7B). Next, we confirmed that IL1β was expressed around infected cells. IL1β was mostly expressed nearby infected cells (Fig. 7C). TNFα was expressed around Aβ^+^ cells in infected fCBOs (Fig. S5B). COs and BVOs also expressed IL1β and TNFα after infection, but the expression levels were lower than fCBOs (Figs. 7C, S5A, B). Moreover, microglia expressed iNOS, which is expressed by M1 activated microglia (Fig. 7D). IL18 is produced by activated microglia and reactive astrocytes in AD [41] and activated microglia can induce the transformation of astrocytes into the A1 phenotype by releasing IL1α [42]. These activated glia-related cytokine genes were increased relative to mock control whereas COs and BVOs, which were restricted in glial expressions, showed lower increase after infection (Fig. S5C). We confirmed that IL18 was expressed around the activated microglia in infected fCBOs (Fig. S5D). Furthermore, C3 expressing reactive astrocytes were observed in infected fCBOs (Fig. 7E). To confirm that proinflammatory cytokines which upregulated by SARS-COV-2 infection promoted Aβ deposition, fCBOs were treated with TNFα, IL1β and IL6 for 4 days. We observed that upregulation of glia and pTau expression, Aβ accumulation including Aβ42 and decreased TUJ1 expression (Fig. S6A, B). Together, these results demonstrated that proinflammatory cytokines released by SARS-CoV-2 infected cells induced activation of glia, which promoted Aβ deposition that induce AD phenotypes in fCBOs.

## Discussion

Recent clinical studies have reported that SARS-CoV-2 causes neurological manifestations by neuroinvasion via blood brain barrier disruption [43]. For instance, an increase of TGFβ and oxidative stress were associated with pTau pathogenesis, in the postmortem COVID-19 patients’ brains [16]. Most studies have been conducted using post-mortem brain tissues or cerebrospinal fluid, which has a limitation to exploring ongoing pathology. Therefore, iPSC-derived brain organoids have been employed as an alternative model to investigate SARS-CoV-2 neurotropism [19, 20, 24, 26, 30]. However, even these brain organoid models have limitations, including lack of immune cells and vascular networks, as they do not sufficiently reproduce such pathological phenomena. To overcome such limitations, we attempted to generate vascularized brain organoids. Given that ACE2 is specifically and highly expressed in microvascular pericytes [44], BVOs consisting of pericytes and ECs were fused into COs to provide vascular networks as well as viral receptors. Hence, fCBO could recapitulate SARS-CoV-2 pathology owing high infection rate. One study confirmed that SARS-CoV-2 productively infects COs containing pericyte-like cells but not COs [25].

We noted that SARS-CoV-2 infected fCBOs exhibits inflammatory responses including activation of endothelial cells and secretion of proinflammatory cytokines. In general, due to the loosening of tight junction induced by cytokines [45], it was expected that vascular leakage process facilitates viral entrance into the brain parenchyma via blood bran barrier [46]. Recently, it was reported that SARS-CoV-2 crosses the blood-brain barrier accompanied with basement membrane disruption without tight junction alteration in vivo [47]. SARS-CoV-2, which entered the brain through the blood-brain barrier, causes inflammation in neuronal cells. According to the results of our study, due to the high SARS-CoV-2 infection rate of fCBOs, the inflammation response following infection was severe than COs. In addition, astrocytes and microglia present in fCBOs further exacerbated this inflammation response, thus induced severe pathologies compared to COs. Considering that iPSC-derived pericytes facilitated astrocyte differentiation after incorporation into COs [25], we also verified that pericytes in fCBOs have promoted astrocyte differentiation which provided extracellular matrix, such as Laminin, to COs. As COs undergo neuroectoderm formation by dual-SMAD inhibition [48], mesoderm-derived microglia were rarely expressed. However, via fusion, BVOs provided microglia/macrophages to fCBOs. We confirmed that fCBOs presented CD14^+^CD11b^+^ or CD34^+^ cells and these might be the source of microglial expression. After infection, activated ECs promoted migration of monocytes thereby increasing expression of IBA1^+^ cells in cortical regions.

These glial cells in SARS-CoV-2 infected fCBOs can also contribute to the induction of neurodegenerative diseases such as AD [49]. Fundamentally, AD phenotypes can be induced by viral infection. Recently, the role of Aβ as an antimicrobial peptide after viral infection has been proposed [50]. In one study, herpes simplex virus type 1 catalyzed the aggregation of Aβ42 in vitro and in vivo [51]. Previously, we observed the acceleration of AD phenotypes in brain organoids after Zika virus infection [52]. Besides the direct effect of viral infection to neuron, immune responses followed by infection accelerate Aβ deposition [53], which can be observed in our COs. However, according to the research, brain microvascular injury as well as neuroinflammation by COVID-19 is deeply associated with the progression of the AD phenotype [54]. Therefore, the existence of blood vessels is necessary to the brain organoids to confirm the pathology that occurs during SARS-CoV-2 infection. Notably, microvascular injury, as evidenced by VCAM1 expression, have been linked to relationships between SARS-CoV-2 and AD [54]. Given these, upregulation of VCAM1 expression in our fCBOs after SARS-CoV-2 infection may imply microvascular injury induced by inflammatory responses, which could lead to neuroinflammation. In comparison with COs not including vascular pericytes and glial populations, we observed a severe inflammatory response by cytokines from both activated blood vessels and glia after viral infection in fCBOs. Furthermore, AD-like pathologies, including pTau expression and Aβ42 accumulation, induced by proinflammatory cytokines were confirmed. Proinflammatory cytokines including TNFα, IL1β and IL6 upregulate APP expression [55] and activate BACE1 expression in neuronal cells via NF-κB activation, thereby increasing Aβ production and aggregation [56]. Because of the proinflammatory characteristics of Aβ itself [57], Aβ deposition may exacerbate AD pathology in this vicious cycle following viral infection. Also, increased release of IL1β promotes pTau expression via kinase activation [58, 59].

In this study, we observed that SARS-CoV-2 infection causes neuroinflammation and microvascular injury thereby inducing AD pathologies by utilizing the fusion of COs and BVOs. However, since we analyzed samples by short-term culture (1dpi, 4dpi) of fCBOs infected with high titer of SARS-CoV-2, the time dependent pathological changes of COVID-19 can be further investigated by long-term culture of infected fCBOs. Additionally, due to the link between neuroinflammation and neurodegenerative diseases, other neurodegenerative pathologies may occur after SARS-CoV-2 infection. For instance, in the case of Parkinson’s disease, there is a correlation between the degeneration pathway of dopaminergic neurons due to neuroinflammation mechanisms, such as increased systemic IL6 level [60]. Thus, fCBOs we developed can be used as an advanced in vitro platform to closely recapitulate various neuropathologies caused by SARS-CoV-2 infection.

## Materials/subjects and methods

### Human iPSC lines

Human iPSC line used in this study, KSCBi005-A, was distributed from National Stem Cell Bank of Korea and previously characterized [61].

### Cortical organoid (CO) cultures

We generated COs from hiPSCs as previously described, with modifications [48]. Briefly, hiPSCs were dissociated with dispase (Sigma, MO USA) and seeded in a low-attachment plate to form embryoid bodies in E8 medium (Gibco) with 50 μM Y-27632 (MCE, NJ USA), 50 μM dorsomorphin (Sigma), 50 μl SB-431542 (Tocris, Bristol UK) and incubated for 48 h. After embryonic body formation, the medium was changed daily to E8 medium with 50 μM dorsomorphin, 50 μl SB-431542. From days 6 to 24, the medium was replaced with neural differentiation medium (NDM, neurobasal medium (Gibco), 2% B27 without vitamin A (Gibco), 1% GlutaMax (Gibco)) with 20 ng/ml EGF (Gibco) and 20 ng/ml FGF2 (R&D) and transferred to a low attachment 60 mm dish on a shaking platform. On day 25, the medium was switched to NDM with 20 ng/ml BDNF (Peprotech, NJ USA) and 20 ng/ml NT3 (Peprotech) and changed every other day until day 43. For further maturation, CO was cultured in NDM, and the medium was changed every 3–4 days.

### Blood vessel organoid (BVO) cultures

We generated BVOs from hiPSC as previously described [62]. Briefly, hiPSC aggregates were generated by seeding in a low attachment plate in aggregation medium with 50 μM Y-27632. The next day, the medium was replaced with N2B27 medium supplemented with 12 μM CHIR99021 (Peprotech) and 30 ng/ml BMP4 (Peprotech). Medium was changed every day for 3 days. On day 3, the medium was replaced with N2B27 supplemented 100 ng/ml VEGF-A (R&D Systems, MN USA) and 2 μM forskolin (Sigma). On day 5, aggregates were embedded in Col1-Mat solution and cultured in StemPro-34 SFM medium (Gibco) with 15% FBS, 100 ng/ml VEGF-A and 100 ng/ml FGF2. On day 9, BVN were extracted with 30-gauge needles. After incubation in a low-attachment 60 mm dish overnight, BVNs were transferred to a low-attachment U-bottom 96-well plate to generate self-assembled BVOs and incubated for 5 days.

### Fused cortical-blood vessel organoid (fCBO) cultures

On day 25 of cortical differentiation, COs and BVNs (day 11 of BVO generation) were transferred to a low-attachment U-bottom 96-well plate and fused with each other for 4 days, and the medium was changed every day. On day 29, fCBOs were transferred to a low attachment 24-well plate with cutting tips, and the medium was changed every other day. On day 43, fCBOs were transferred to a low-attachment 60 mm dish on a shaking platform, and the medium was changed every 3–4 days. The medium was CO medium mixed with BVO medium at a 9:1 ratio.

### Organoid infection experiments

Organoids (fCBOs and COs on day 57) were transferred to a low-attachment 24-well plate with single organoids per well. Infections were performed by dilution of the virus in culture medium at a titer of 2 × 10^6^ TCID_50_/ml. The medium was removed and replaced with fresh organoid culture medium.

### Quantification and statistical analysis

All data were reported as the mean ± SEM. The statistical analyses were performed using GraphPad Prism version 9.0 (GraphPad Software, CA USA). The number of replicates is indicated in figure legends. For statistical comparisons, data were analyzed by Student’s *t*-test using the *p*-values: ***p < 0.001, **p < 0.01, *p < 0.05. The number of biological replicates is specified in the figure legends. Sample was randomly selected. Mean fluorescence intensities (MFIs) was measured in the segmented organoid regions. Cell numbers were quantified manually using the cell counter function in ImageJ (NIH).

## Supplementary information

Supplementary Information

Original Data File

## Data Availability

The data that support the findings are available from the Lead Contact, Kyung-Sun Kang (kangpub@snu.ac.kr), upon request.
