# Supplementary material for: Cortical-blood vessel assembloids exhibit Alzheimer’s disease phenotypes by activating glia after SARS-CoV-2 infection
Source: Cell Death Discov. 2023 Jan 25;9:32. doi: 10.1038/s41420-022-01288-8 (PMC9876421; doi:10.1038/s41420-022-01288-8)
Supplement: Supplementary file 1 — Supplementary Information [file 41420_2022_1288_MOESM1_ESM.docx]

**Supplementary Information**

**Supplementary Figures**


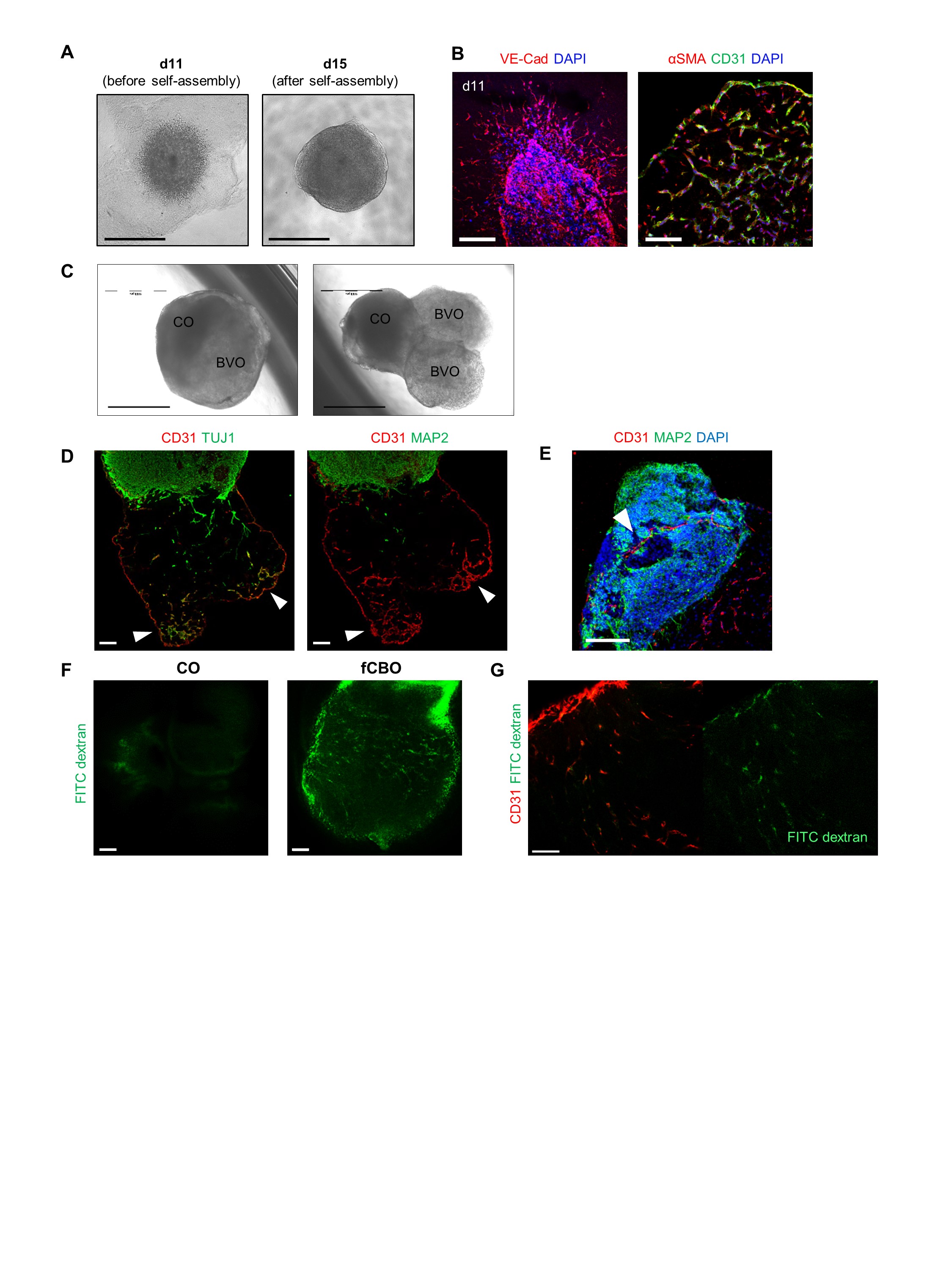


**Fig. S1 Additional characterization of fused cortical-blood vessel organoids, cortical organoids and blood vessel organoids, related to Fig. 1. *A*** Bright-field image of blood vessel organoids on day 11 (left) and day 15 (right). Scale bars = 1mm. ***B*** Left, immunostaining of BVOs for CD31 and vascular smooth muscle cells (vSMCs) marker αSMA on day 15. Right, Whole-mount staining of BVOs for endothelial marker VEGFR2 and basement membrane marker collagen type 4 (Col4). BVOs showed self-assembled blood vessels (arrow) and formed 3D blood vessel structures with endothelial tubes surrounded by vSMCs and basement membrane. Scale bars = 100 μm. ***C*** Left, bright-field image of 1:1 fCBOs on day 43. Right, bright-field image of 1:2 fCBOs on day 43. Scale bars = 1 mm. ***D*** Immunostaining of fCBOs for CD31 and TUJ1 or MAP2. TUJ1+ MAP2- newly generated neurites (arrows) on day 57. ***E*** Immunostaining of fCBOs for CD31 and MAP2. CD31+ blood vessels penetrated cortical region of fCBOs (arrow) on day 57. Scale bar = 100 μm. ***F*** Whole-mount staining after 1 hr FITC-dextran perfusion of COs and fCBOs. Scale bars = 100 μm. ***G*** Immunostaining of fCBOs after 1 hr FITC-dextran perfusion for CD31. Scale bar = 100 μm.


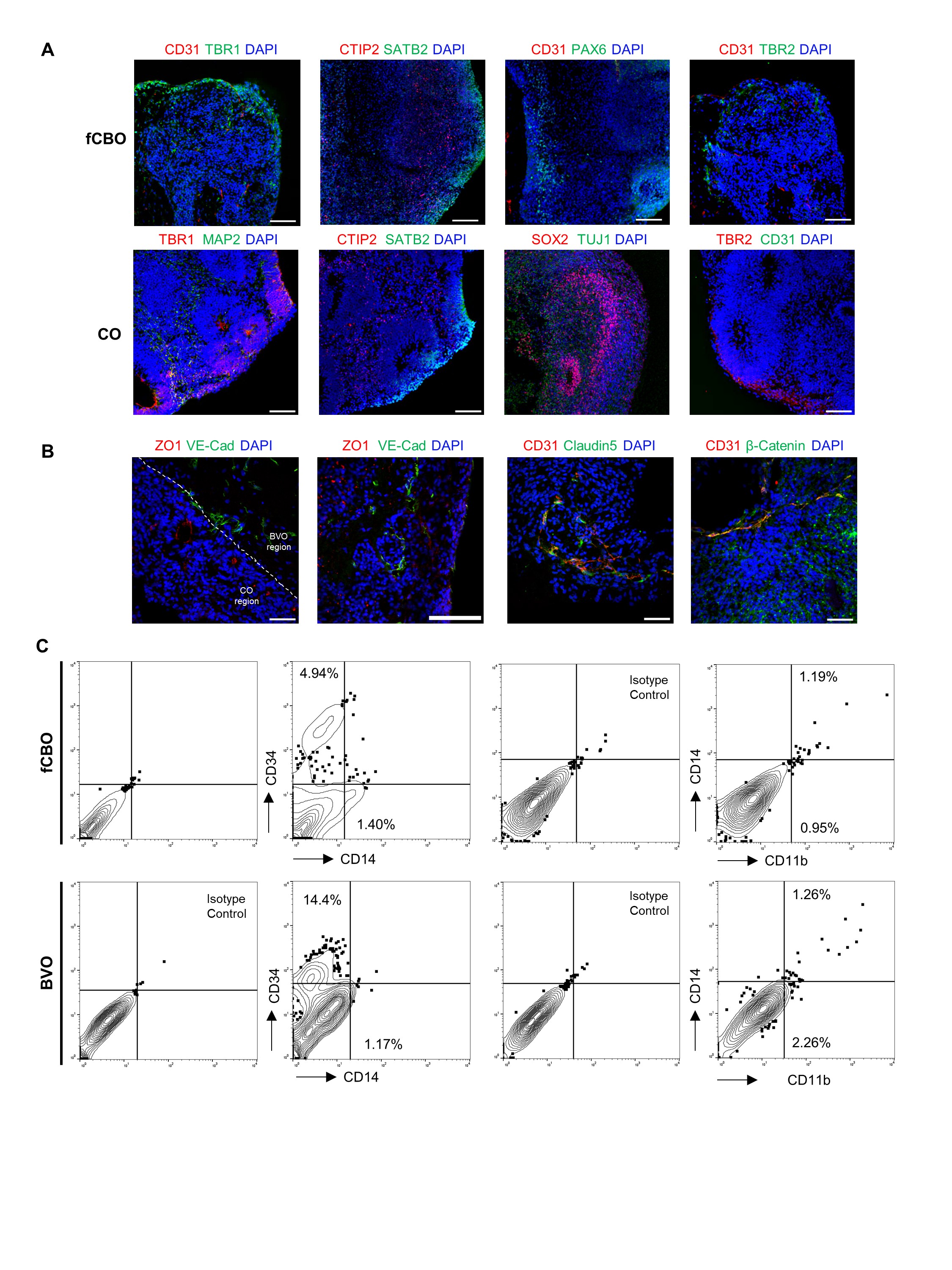


**Fig. S2 Additional characterization of fused cortical-blood vessel organoids, cortical organoids and blood vessel organoids, related to Fig. 2.**

***A*** Immunostaining of fCBOs and COs for cortical marker TBR1, CTIP2, SATB2, PAX6, TBR2, MAP, TUJ1 and CD31 on day 57. Scale bars = 100 μm. ***B*** Immunostaining of fCBOs for tight junction marker ZO1, Claudin-5 and adherens junction marker β-catenin, VE-cadherin and CD31 on day 57. VE-Cad; VE-cadherin. Scale bars = 100 μm. ***C*** Representative contour plot images of FACS analysis for CD14, CD34, CD11b expression in BVOs (day 15) and fCBOs (day 57).


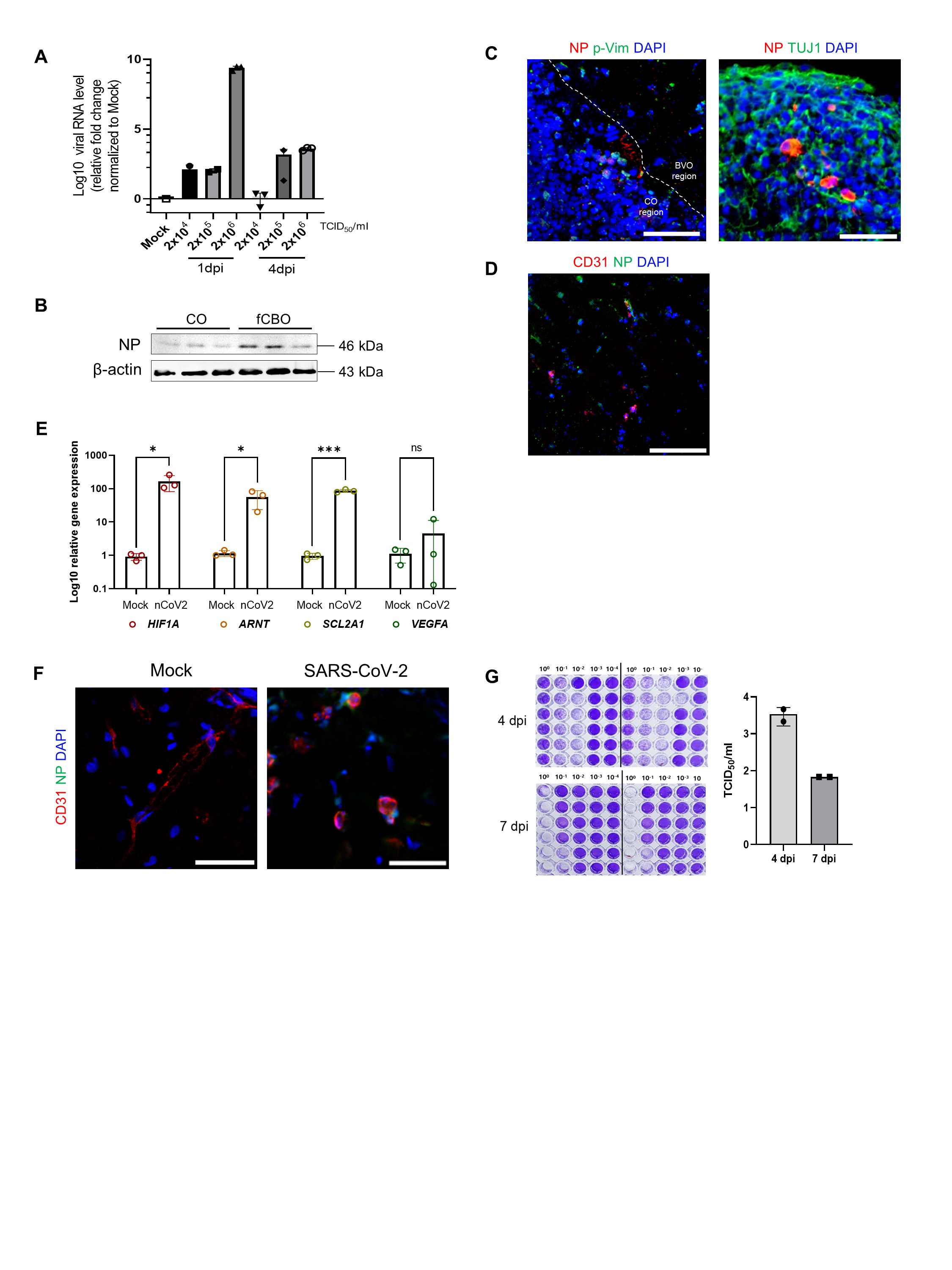


**Fig. S3 Additional characterization of SARS-CoV-2 infected fused cortical-blood vessel organoids, related to Figure 3.**

***A*** Relative viral RNA levels for N1 in viral titers of 2X10^4^, 2X10^5^ and 2X10^6^ TCID_50_/ml at 1 dpi and 4 dpi to mock infected control. Values represent mean ± SEM with individual data points plotted (n = 3 organoids per batch, 1 batch). ***B*** Western blot for SARS-CoV-2 nucleoprotein (NP) and the loading control β-actin of SARS-CoV-2 infected COs and fCBOs (n = 3 organoids). ***C*** Immunostaining of SARS-CoV-2 infected fCBOs for NP and radial glial marker phospho-vimentin (pVim) in VZ (left). Immunostaining of SARS-CoV-2 infected fCBOs for NP and TUJ1 in superficial layer (right). Scale bars = 100 μm. ***D*** Immunostaining of SARS-CoV-2 infected fCBOs for NP and CD31 in blood vessel organoid region. Scale bar = 100 μm. ***E*** Relative expression of Hif1α pathway genes HIF1A, ARNT, SCL2A1 and VEGFA of SARS-CoV-2 or mock infected fCBOs by qRT-PCR. Values represent mean ± SEM with individual data points plotted (n = 3 organoids per batch, 1 batch). ***F*** Immunostaining of SARS-CoV-2 or mock infected fCBOs for NP and CD31. Scale bars = 100 μm. ***G***Titration of budded virus at 4 dpi and 7 dpi of SARS-CoV-2 infected fCBOs. Titer calculation was performed using Spearman-Karber method. Values represent mean ± SEM with individual data points plotted (n = 2 organoids per batch, 1 batch).


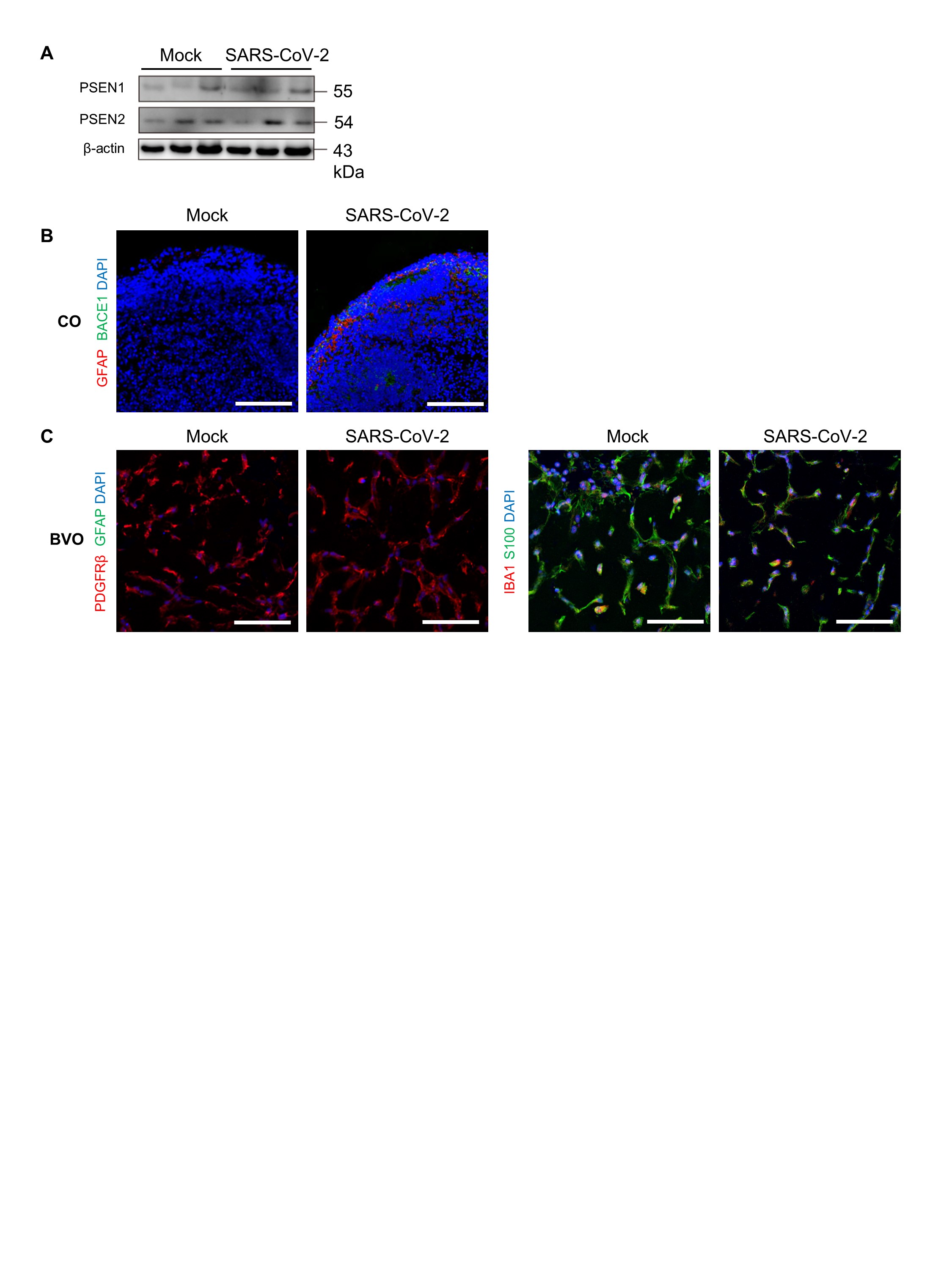


**Fig. S4 Additional characterization of Alzheimer’s disease pathologies of SARS-CoV-2 infected fused cortical-blood vessel organoids, related to Figure 4-6.**

***A*** Western blot for PSEN1, PSEN2 and the loading control β-actin of SARS-CoV-2 or mock infected fCBOs (n = 3 organoids per batch, 1 batch). ***B*** Immunostaining of SARS-CoV-2 or mock infected COs for GFAP and BACE1. Scale bars = 100 μm. ***C*** Immunostaining of SARS-CoV-2 or mock infected BVOs for PDGFRβ and GFAP, IBA1 and S100. Scale bars = 50 μm.


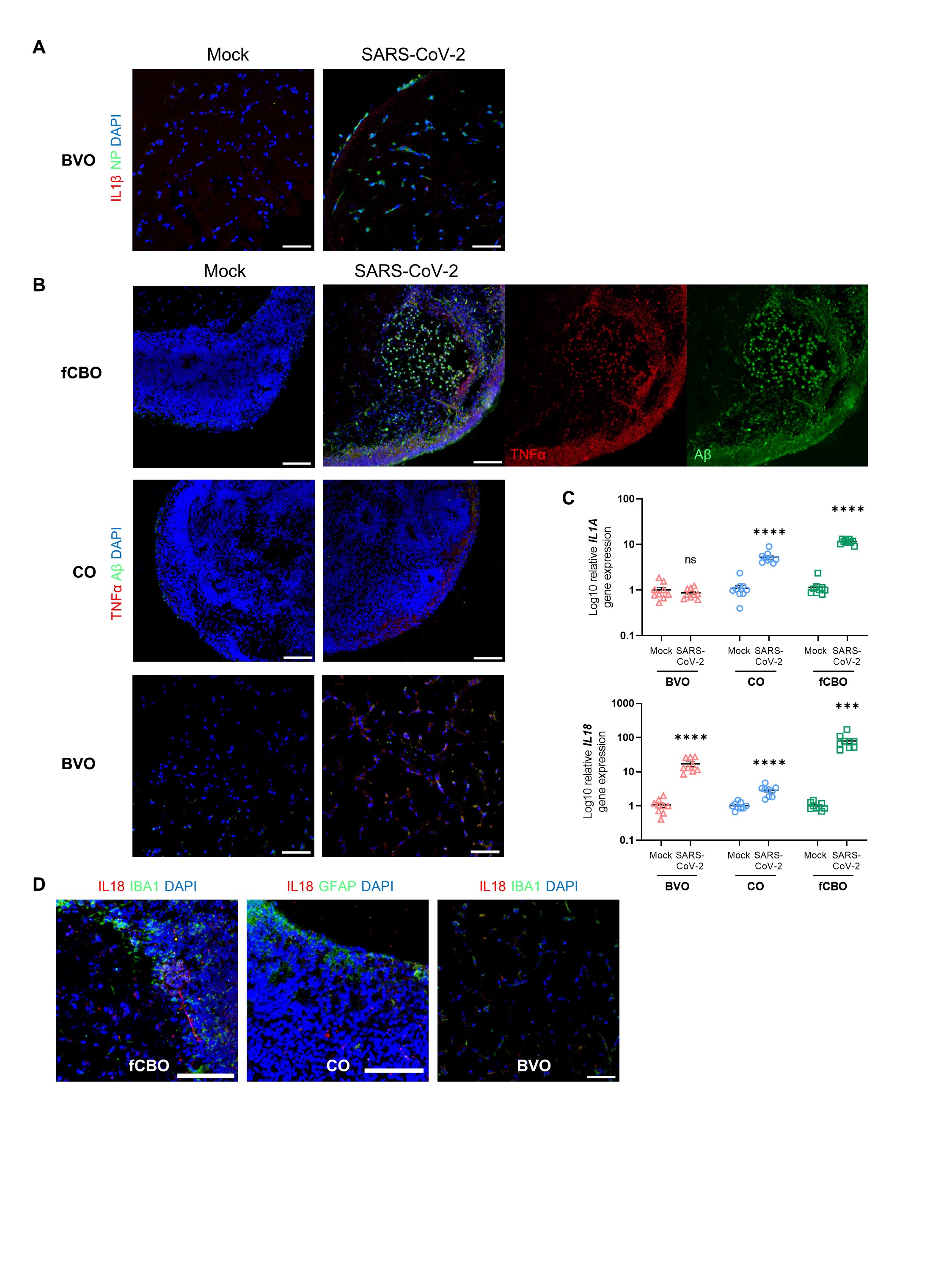


**Fig. S5 Additional characterization of released proinflammatory cytokines and activated glial cells in SARS-CoV-2 infected fused cortical-blood vessel organoids, cortical organoids and blood vessel organoids, related to Figure 6.**

***A*** Immunostaining of SARS-CoV-2 or mock infected BVOs for IL1β and SARS-CoV-2 NP. Scale bars = 100 μm. ***B*** Immunostaining of SARS-CoV-2 or mock infected fCBOs, COs and BVOs for TNFα and Aβ. Scale bars = 100 μm. ***C*** Western blot for SARS-CoV-2 nucleoprotein (NP) and the loading control β-actin of SARS-CoV-2 infected COs and fCBOs (n = 3 organoids). ***C*** Relative gene expression of SARS-CoV-2 or mock infected fCBOs, COs and BVOs for IL18 and IL1A. Values represent mean ± SEM with individual data points plotted (n = 3 organoids per batch, 3 batches). ***D*** Immunostaining of SARS-CoV-2 infected fCBOs, COs and BVOs for IL18 and IBA1 or GFAP. Scale bars = 100 μm.


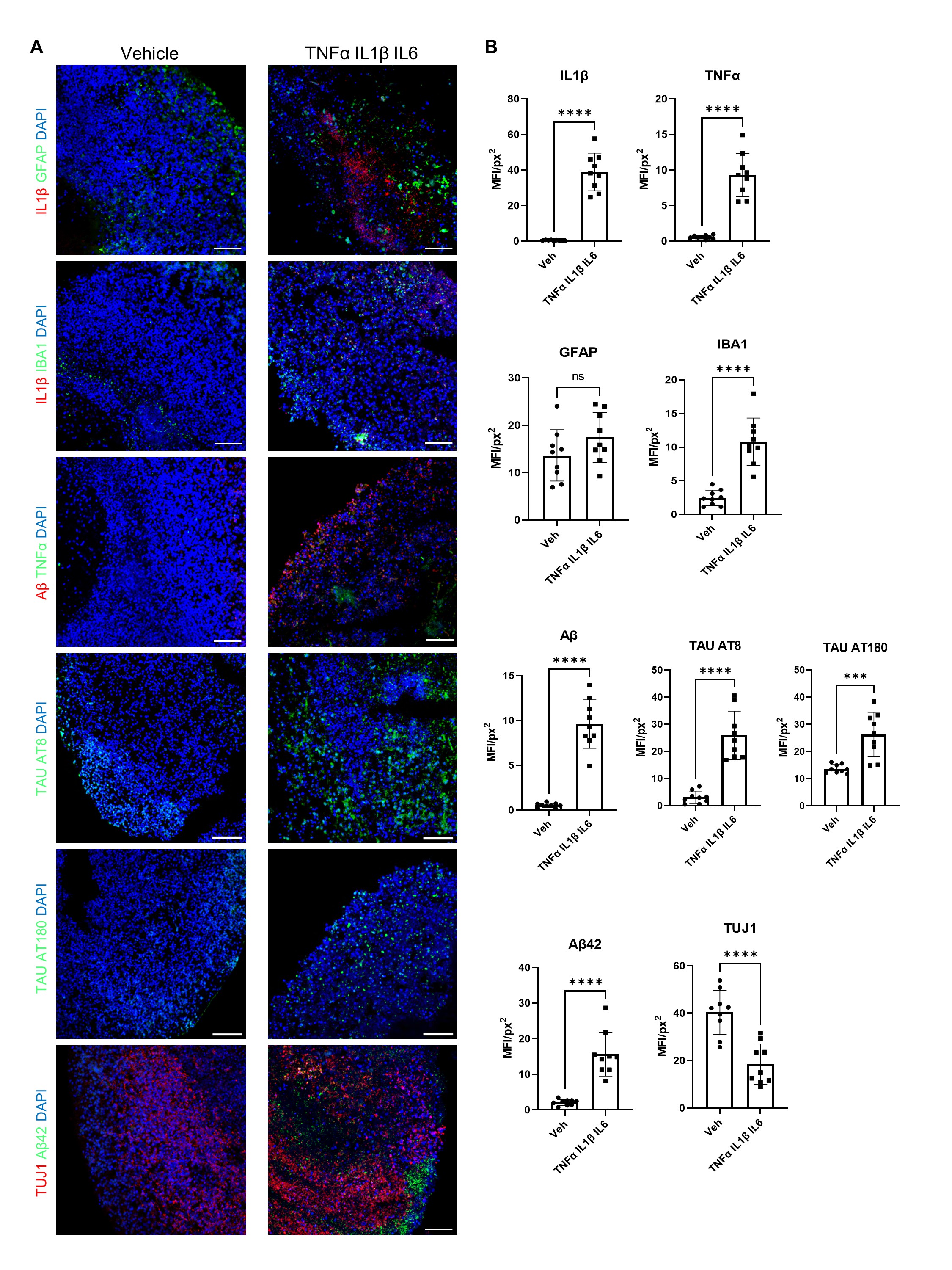


**Fig. S6 Proinflammatory cytokine treated fused cortical-blood vessel organoids exhibits Alzheimer’s disease pathologies including phosphorylated tau and β-amyloid accumulation.**

***A*** Immunostaining of vehicle or TNFα, IL1β and IL6 treated fCBOs for IL1β and GFAP or IBA1, TNFα and Aβ, AT180, AT8, TUJ1 and TNFα. Scale bars = 100 μm. ***B*** Quantification of fluorescence expression levels of vehicle or TNFα, IL1β and IL6 treated fCBOs for IL1β, TNFα, GFAP, IBA1, Aβ, AT180, AT8, Aβ42 and TUJ1. Values represent mean ± SEM with individual data points plotted (n = 3 organoids per batch, 3 batches).

**Methods**

Preparation of viruses

SARS-CoV-2 was obtained from National Culture Collection for Pathogens and amplified in the Vero-E6 cell line. Briefly, one day before the infection, 8×10^6^ cells of the Vero-E6 were seeded into the T75 flask. Before infection, the media has been removed without disrupting the cell monolayer and added 10 ml of fresh media. And then Vero-E6 cells were infected with SARS-CoV-2. After three days of infection, the culture media (DMEM (Hyclone, MA USA) containing 10% FBS (Gibco, NY USA) and 1% P/S (Gibco)) were harvested and centrifuged for 3,000 rpm at 10 minutes for the removal of cell debris. The Harvested viruses were stored at -80℃. Infectious virus titers were determined based on the 50% tissue culture infectious dose (TCID_50_) according to the endpoint method by Spearman-Karber.

FITC-dextran perfusion

*In vitro* perfusion was performed as previously described [69]. Briefly, organoids were located between two syringe filters (PALL, NY USA) which were attached to silicon tube and perfused for 1 h under constant flow rate of 1.5ml/min in 37℃ incubator using a peristaltic infusion pump.

qRT-PCR analysis

RNA was extracted with Trizol reagent (Invitrogen, MA USA). Complementary DNA was synthesized using Superscript reverse transcriptase (Invitrogen). Quantitative real-time PCR was performed using the SYBR Green PCR Master Mix (Applied Biosystems, MA USA).

Western blot analysis

Lysates were prepared from each organoid with Pro-prep lysis buffer (Intron Biotechnology, Seongnam ROK). The proteins were separated via 10 and 15% SDS polyacrylamide gel electrophoresis and transferred to nitrocellulose membranes. After blocking with 3% bovine serum albumin solution, the proteins on the membrane were incubated with primary antibodies overnight at 4°C. The secondary antibodies used were horseradish peroxidase-conjugated antibodies (Invitrogen). The protein and antibody complexes were detected using an enhanced chemiluminescence detection kit (GE Healthcare Life Science, MA USA) and analyzed.

Immunostaining

Each organoid was fixed with 4% PFA overnight at 4°C. The fixed sample was incubated in 30% sucrose buffer at 4°C for one day. Organoids were embedded in plastic cryomold with gelatin solution and frozen on liquified nitrogen. Serial tissue sections were sliced using a cryostat. For immunofluorescence staining, the tissue sections were outlined with a hydrophobic pen (Dako, Glostrup Denmark) and washed with PBS to clear extra gelatin at 37°C water bath. Tissue sections were permeabilized and blocked for 1 h at room temperature using a blocking buffer (PBS with 3% FBS, 1% BSA (GenDepot, TX USA), 0.5% Triton X-100 (Sigma), 0.5% Tween 20 (Amresco, TX USA) and 0.01% (w/v) sodium deoxycholate solution) and incubated with primary antibodies (Supplementary Table 1) diluted in blocking solution overnight at 4°C. After washing in PBS-T solution (PBS with 0.05% Tween 20) 3 times, tissue sections were incubated with secondary antibodies (Supplementary Table 1) diluted in blocking buffer for 1h at room temperature. After washing in PBS-T solution 2 times, tissue sections were incubated with DAPI solution (1:1000 in PBS) for 10 min. After washing in PBS, slides were mounted using mounting medium (Dako). For whole-mount staining of BVO, we performed staining as previously described [68]. Briefly, fixed organoids were added blocking buffer for 2 h at room temperature and incubated in primary antibody diluted in blocking buffer for overnight on a rocking shaker at 4°C. After washed in PBS-T solution on an orbital shaker, organoids were added secondary antibodies in blocking buffer and incubated for 2h at room temperature. After washing in PBS-T solution, organoids were incubated in DAPI solution for 10min and mounted on 22x50-mm coverslip.

TCID_50_ test for progeny viruses

Organoids were infected by 2×10^6^ TCID_50_ of SARS-CoV-2, respectively. After 1 day incubation, viruses were removed, and fresh organoid culture media were added. And then, culture media were harvested after additional 3 days and 7 days incubation. Harvested culture media were pooled, and 10-fold diluted 100 to 10-8 serially. 1 day before the TCID_50_ test, Vero-E6 cells were seed to the 96-well cell culture plate and each diluent was added to Vero-E6 cells. After 3 days incubation, cells were stained by crystal violet solution.

Flow cytometry analysis of organoids

Organoids were mechanically disrupted and disaggregated using 3U/ml dispase (Sigma), 2U/ml collagenase type 1 (Sigma) and 100U DNase 1 (Sigma) in PBS for 20 min at 37℃. Single cells were stained with fluorescence conjugated antibodies (Supplementary Table 1). A BD FACS Calibur was used for cell analysis.

ELISA quantification

The Aβ42, Aβ40, TNF, IL1β and IL6 levels in RIPA were measured using Human β-Amyloid (1-42) ELISA kit (Invitrogen), Human β-Amyloid (1-42) ELISA kit (Invitrogen), Human TNF-α ELISA kit (R&D systems), Human IL-1β/IL-1F2 ELISA kit (R&D systems) and Human IL-6 ELISA kit (R&D systems) according to the manufacturer’s instructions. The reaction was read at 450nm with microplate reader (Tecan).

**Supplementary Table**

Supplementary Table 1. List of antibodies used in this study.

| **Name** | **Vendor** | **Catalog #** |
| --- | --- | --- |
| FITC Mouse anti-CD14 | BD Biosciences | Cat# 555397, RRID:AB_395798 |
| FITC Mouse anti-CD45 | BD Biosciences | Cat# MAB933, RRID:AB_2223153 |
| Mouse anti-ACE2 | R&D Systems | Cat# MAB933, RRID: AB_2223153 |
| Mouse anti-BACE1 | R&D Systems | Cat# MAB931, RRID: AB_2258772 |
| Mouse anti-VE-Cad | BD Biosciences | Cat# 555661, RRID: AB_396015 |
| Mouse anti-CD31 | Invitrogen | Cat# 14-0319-82, RRID: AB_467204 |
| Mouse anti-Cloudin5 | Invitrogen | Cat# 35-2500, RRID: AB_2533200 |
| Mouse anti-GFAP | Cell signaling technology | Cat# 3670, RRID: AB_561049 |
| Mouse anti-HIF1a | Novus biologicals | Cat# NB 100-105, RRID: AB_350048 |
| Mouse anti-IBA1 | Abcam | Cat# ab15690, RRID:AB_2224403 |
| Mouse anti-ICAM1 | Santa Cruz Biotechnology | Cat# sc-107, RRID: AB_627120 |
| Mouse anti-MAP2 | Merck | Cat# MAB3418, RRID: AB_94856 |
| Mouse anti-PDGFRß | R&D Systems | Cat# MAB1263, RRID: AB_2162792 |
| Mouse anti-TAU AT8 | Invitrogen | Cat# MN1020, RRID: AB_223647 |
| Mouse anti-TAU AT180 | Invitrogen | Cat# MN1040, RRID: AB_223649 |
| Mouse anti-p-vimentin (pS55) | MBL International | Cat# D076-3S, RRID: AB_592962 |
| Mouse anti-S100 | Santa Cruz Biotechnology | Cat# sc-56477 |
| Mouse anti-SATB2 | Abcam | Cat# ab92446; RRID: AB_10563678 |
| Mouse anti-ß amyloid | Biolegend | Cat# 803001, RRID: AB_2564653 |
| Mouse anti-ß-actin | Cell signaling technology | Cat# 4967, RRID: AB_330288 |
| Mouse anti-ß-catenin | Millipore | Cat# 05-613, RRID: AB_11213062 |
| Mouse anti-TUJ1 | Biolegend | Cat# 801202, RRID: AB_10063408 |
| PE Mouse anti-CD34 | BD Biosciences | Cat# 555822, RRID: AB_396151 |
| PE Mouse anti-IL1β | Invitrogen | Cat# 12-7018-41, RRID: AB_11218702 |
| PE Rat anti-CD11b | BD Biosciences | Cat# 553311, RRID: AB_394775 |
| Rabbit anti-C3 | Cell signaling technology | Cat# 97425S |
| Rabbit anti-CD31 | Abcam | Cat# ab28364, RRID: AB_726362 |
| Rabbit anti-GFAP | Dako | Cat# N1506, RRID: AB_10013482 |
| Rabbit anti-IBA1 | Wako | Cat# 019-19741, RRID: AB_839504 |
| Rabbit anti-IL18 | R&D Systems | Cat# MAB91243 |
| Rabbit anti-iNOS | Abcam | Cat# ab15323, RRID: AB_301857 |
| Rabbit anti-PAX6 | Biolegend | Cat# 901301, RRID: AB_2565003 |
| Rabbit anti-SARS-CoV-2 Nucleoprotein | Sino Biological | Cat# 40143-R001, RRID: AB_2827974 |
| Rabbit anti-ß amyloid | Cell signaling technology | Cat# 14975, RRID: AB_2798672 |
| Rabbit anti-ß amyloid (1-40) | Cell signaling technology | Cat# 12990, RRID: AB_2798082 |
| Rabbit anti-ß amyloid (1-42) | Cell signaling technology | Cat# 12843S, RRID: AB_2798041 |
| Rabbit anti-TBR1 | Abcam | ab31940, RRID: AB_2200219 |
| Rabbit anti-TBR2 | Abcam | Cat# ab23345, RRID:AB_778267 |
| Rabbit anti-TNFa | Abcam | Cat# ab6671, RRID: AB_305641 |
| Rabbit anti-ZO1 | Invitrogen | Cat# 40-2200, RRID: AB_2533456 |
| Rat anti-CTIP2 | Abcam | Cat# ab18465, RRID: AB_2064130 |
| Mouse anti-Laminin b1 | Abcam | Cat# ab8982 |
| Mouse anti-VCAM1 | Santa Cruz Biotechnology | Cat# sc-13160 |
