# Supplementary material for: Cortical-blood vessel assembloids exhibit Alzheimer’s disease phenotypes by activating glia after SARS-CoV-2 infection
Source: Cell Death Discov. 2023 Jan 25;9:32. doi: 10.1038/s41420-022-01288-8 (PMC9876421; doi:10.1038/s41420-022-01288-8)
Supplement: Supplementary file 2 — Original Data File [file 41420_2022_1288_MOESM2_ESM.docx]

Full length WB

(Fig. 2F)


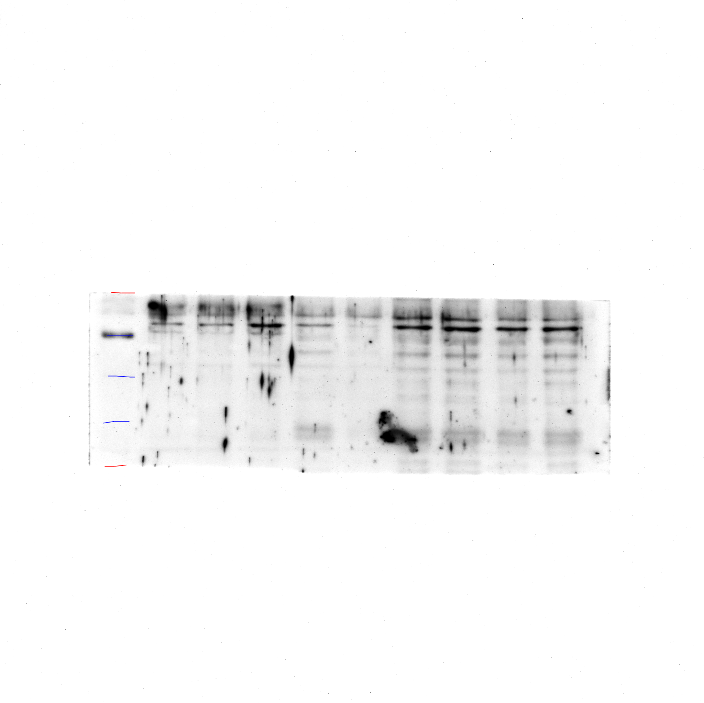


GFAP


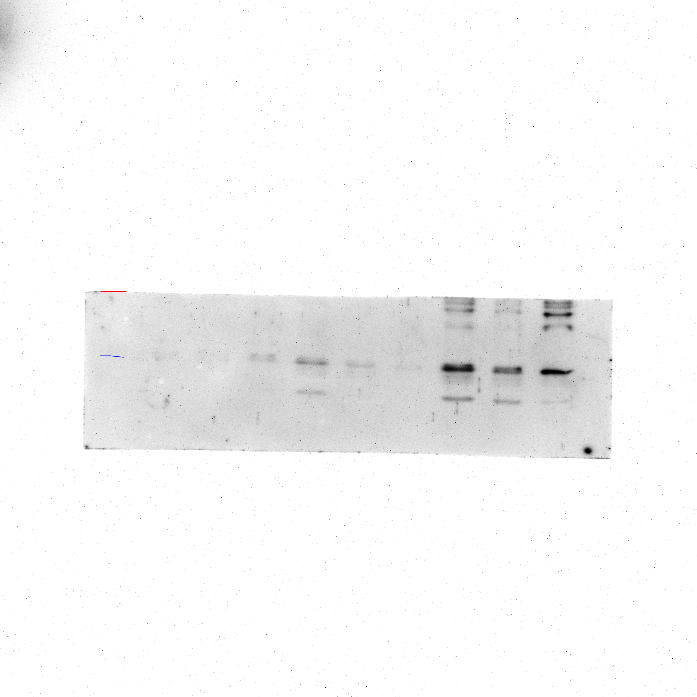


IBA1

b-actin


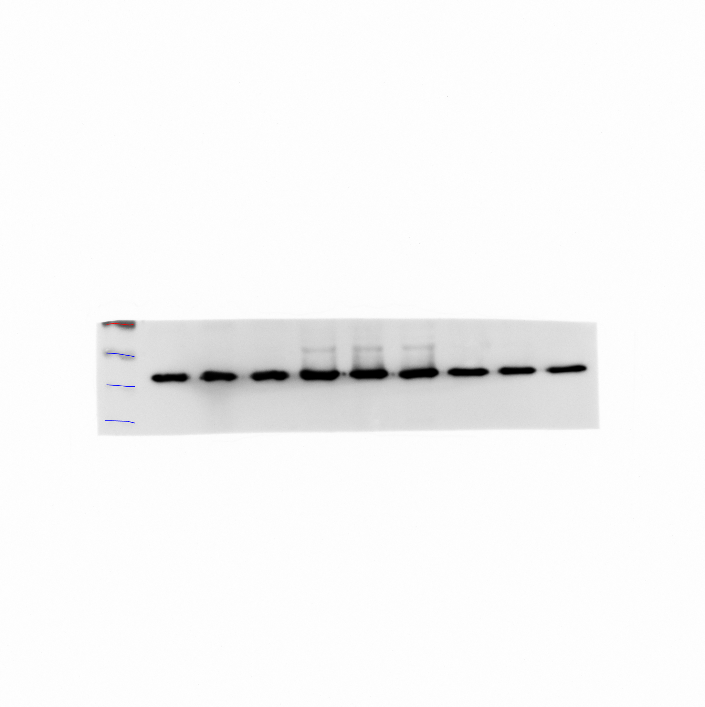


(Fig. 4C)

TAU AT180


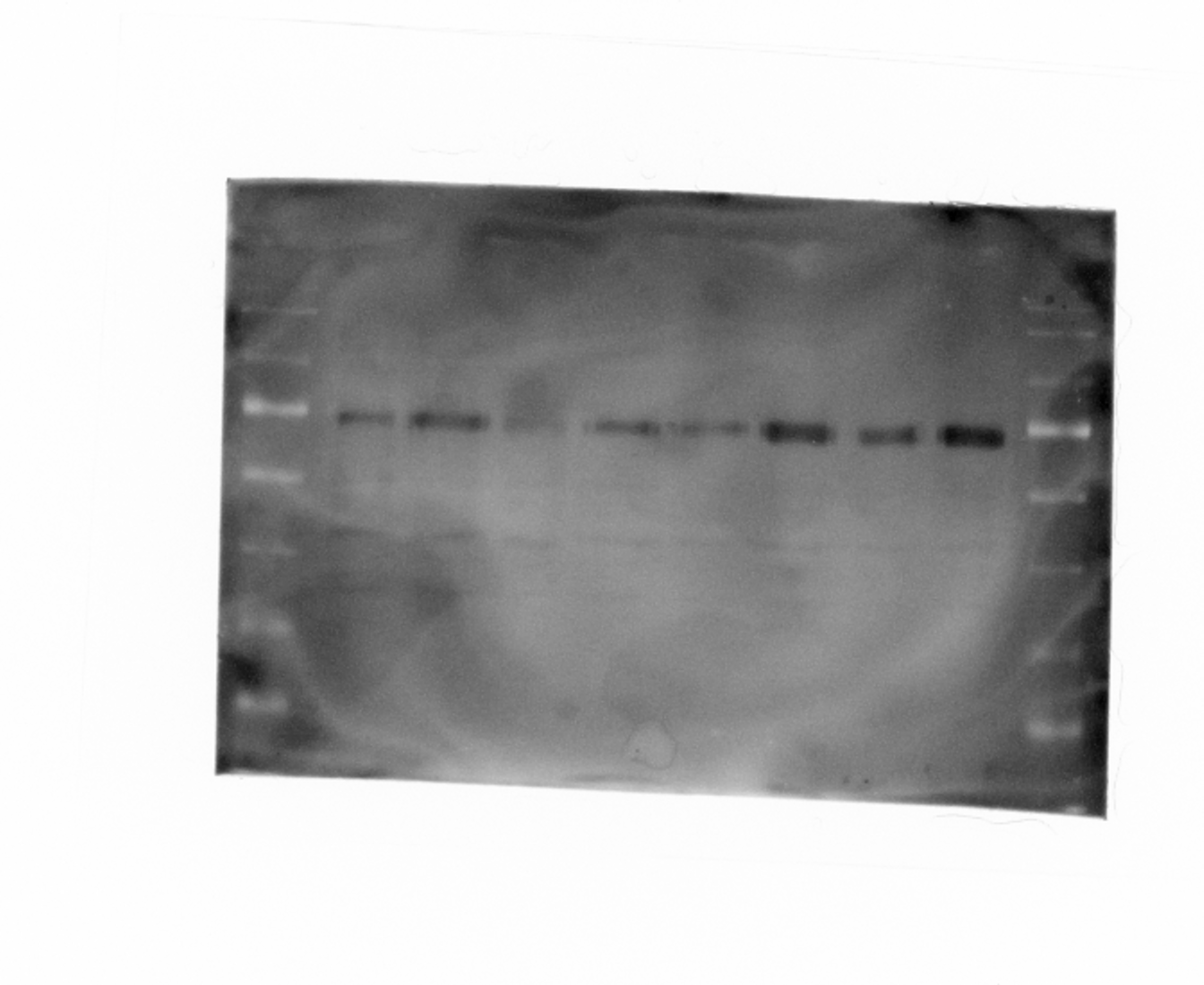


TAU AT8


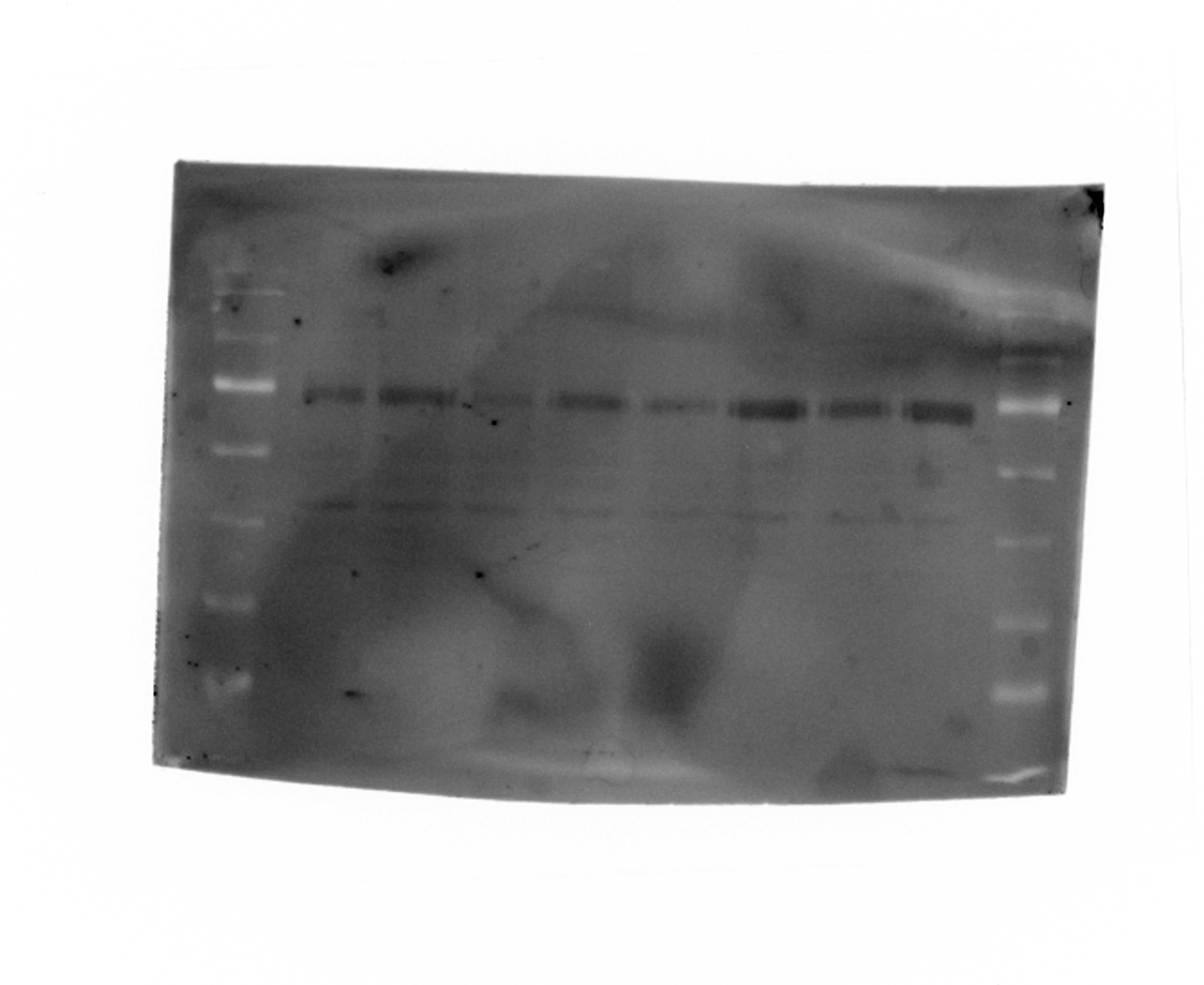





TAU

b-actin


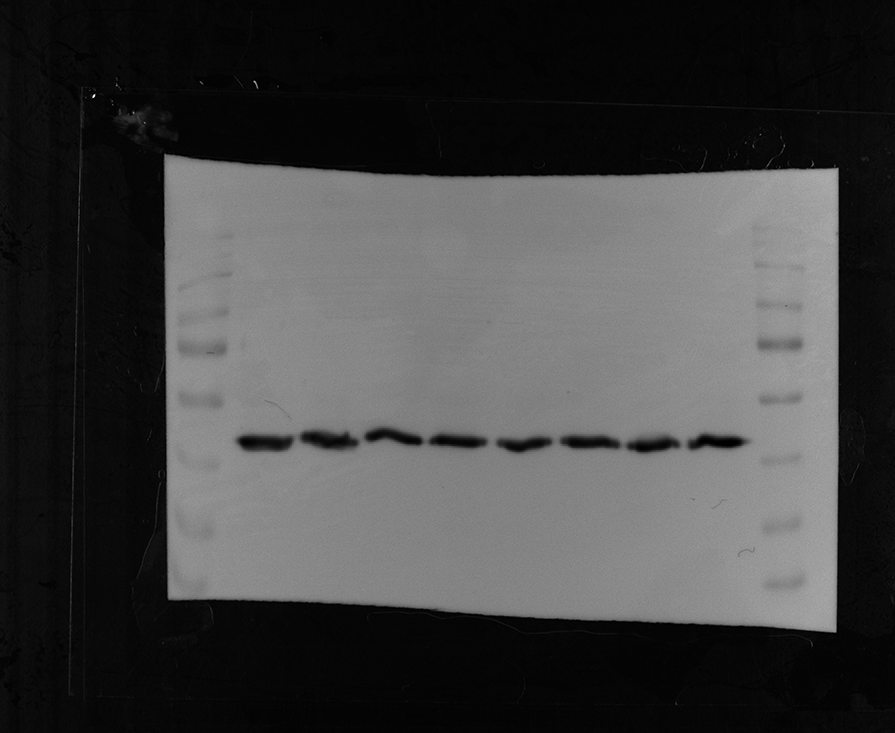


(Fig. 5F)





APP

BACE1


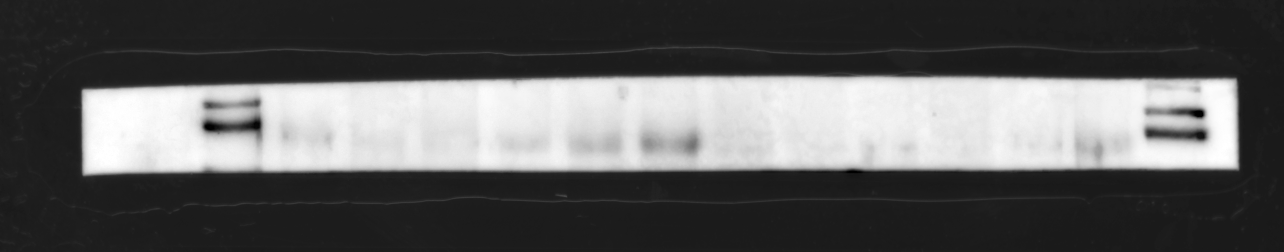





b-actin

(Fig. 6C)


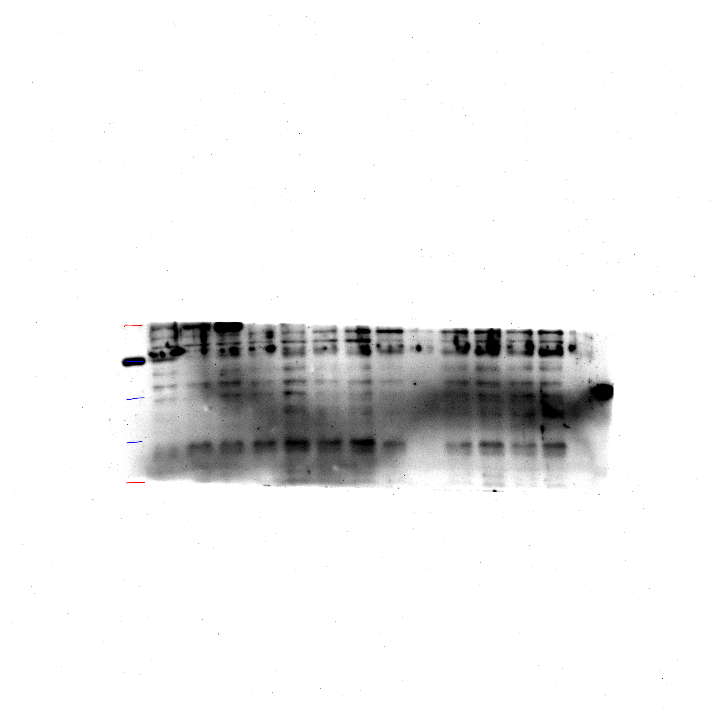


GFAP

IBA1

b-actin


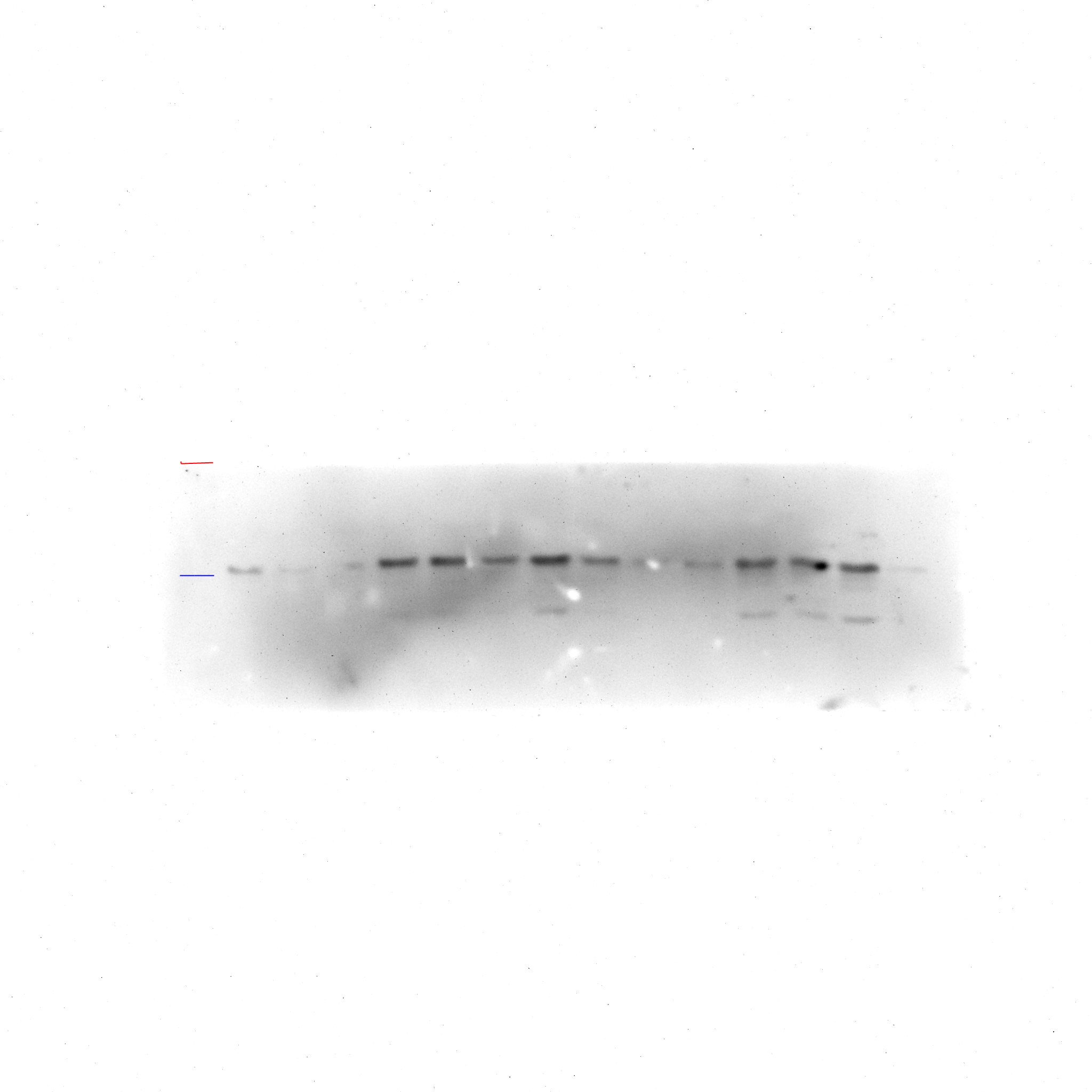

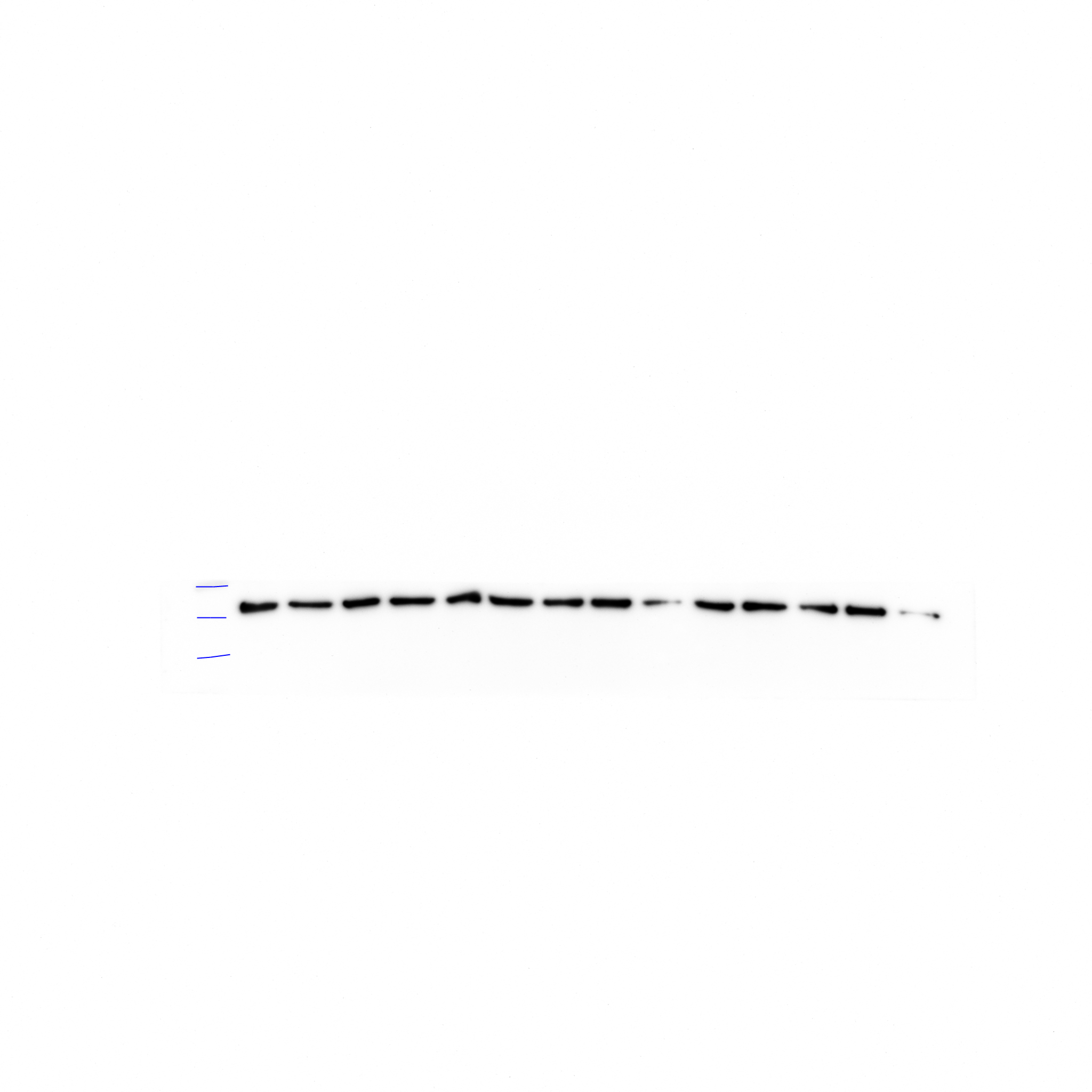


(Fig. S3B)

NP


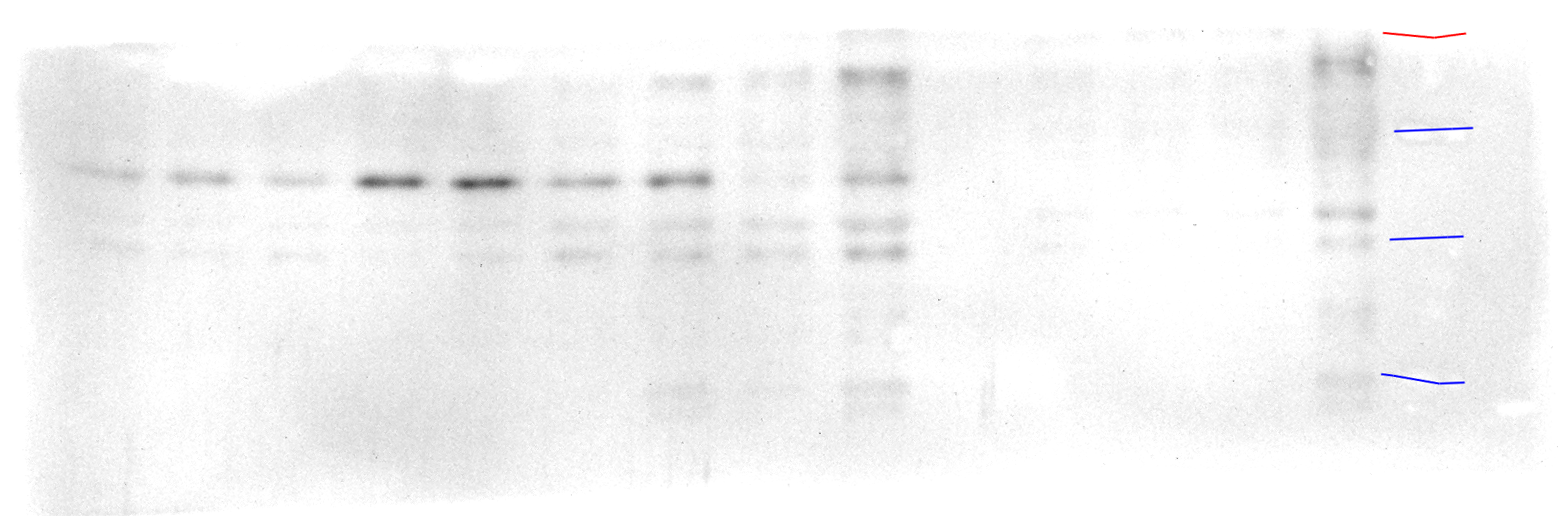


b-actin


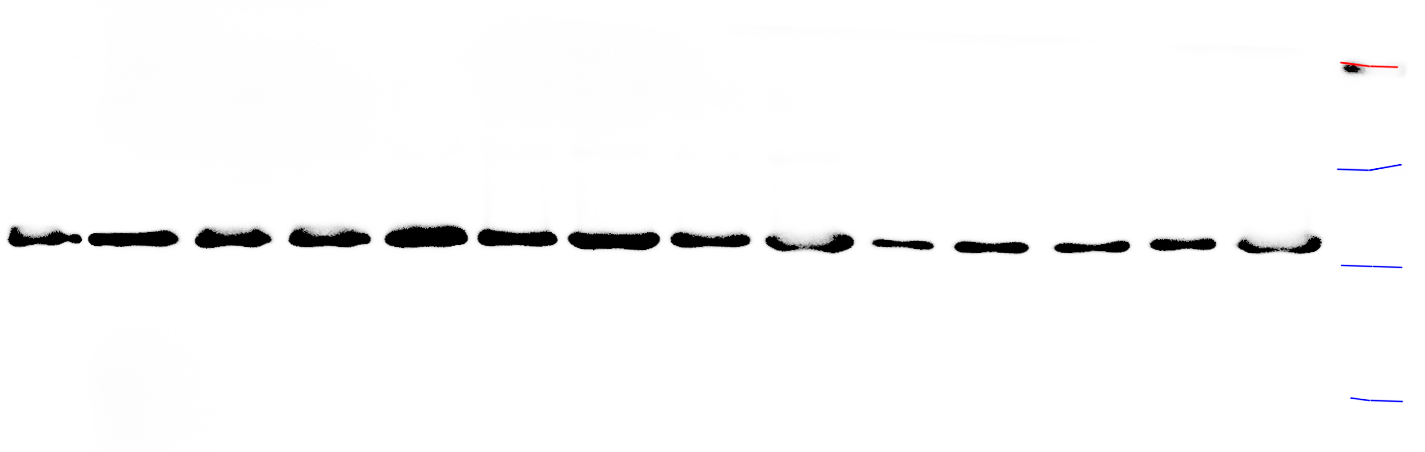


(Fig. S4A)





PSEN1

PSEN2


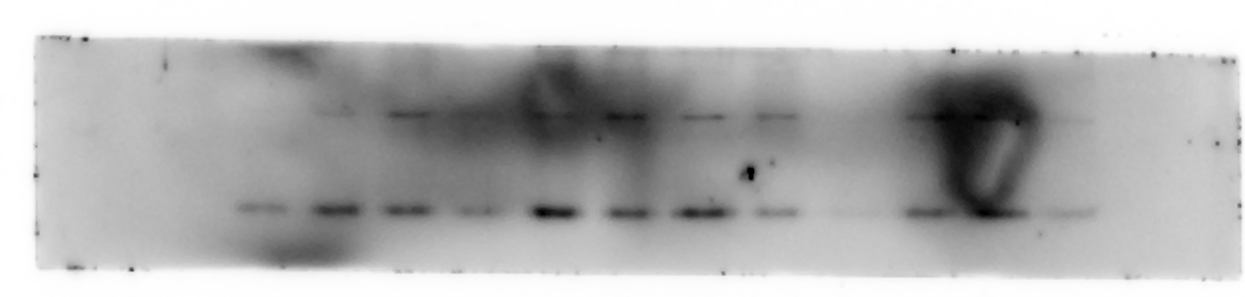





b-actin
